# Integrative analysis identifies an older female-linked AML patient group with better risk in ECOG-ACRIN Cancer Research Group’s clinical trial E3999

**DOI:** 10.1038/s41408-022-00736-z

**Published:** 2022-09-23

**Authors:** Franck Rapaport, Kenneth Seier, Yaseswini Neelamraju, Duane Hassane, Timour Baslan, Daniel T. Gildea, Samuel Haddox, Tak Lee, H. Moses Murdock, Caroline Sheridan, Alexis Thurmond, Ling Wang, Martin Carroll, Larry D. Cripe, Hugo Fernandez, Christopher E. Mason, Elisabeth Paietta, Gail J. Roboz, Zhuoxin Sun, Martin S. Tallman, Yanming Zhang, Mithat Gönen, Ross Levine, Ari M. Melnick, Maria Kleppe, Francine E. Garrett-Bakelman

**Affiliations:** 1 Human Oncology and Pathogenesis Program, Molecular Cancer Medicine Service, Memorial Sloan Kettering Cancer Center, New York, NY USA; 2 Center for Clinical and Translational Science, The Rockefeller University, New York, NY USA; 3 Department of Epidemiology and Biostatistics, Memorial Sloan Kettering Cancer Center, New York, NY USA; 4 Department of Biochemistry and Molecular Genetics, University of Virginia, Charlottesville, VA USA; 5 Division of Hematology and Medical Oncology, Department of Medicine, Weill Cornell Medicine, New York, NY USA; 6 Cancer Biology and Genetics Program, Sloan Kettering Institute, Memorial Sloan Kettering Cancer Center, New York, NY USA; 7 Division of Hematology and Oncology, University of Pennsylvania Perelman School of Medicine, Philadelphia, PA USA; 8 Simon Cancer Center, Indiana University, Indianapolis, IN USA; 9 Department of Malignant Hematology & Cellular Therapy, Moffitt Cancer Center, Tampa, FL USA; 10 Department of Physiology and Biophysics, Weill Cornell Medicine, New York, NY USA; 11 Institute for Computational Biomedicine, Weill Cornell Medicine, New York, NY USA; 12 The WorldQuant Initiative for Quantitative Prediction, Weill Cornell Medicine, New York, USA; 13 Montefiore Medical Center, Bronx, NY USA; 14 Weill Cornell Medicine and The New York Presbyterian Hospital, New York, NY USA; 15 Dana-Farber Cancer Institute, Boston, MA USA; 16 Memorial Sloan Kettering Cancer Center, New York, NY USA; 17 Department of Pathology, Memorial Sloan Kettering Cancer Center, New York, NY USA; 18 Department of Medicine, University of Virginia, Charlottesville, VA USA; 19 University of Virginia Cancer Center, Charlottesville, VA USA

**Keywords:** Cancer genetics, Cytogenetics, Cancer genetics

## Abstract

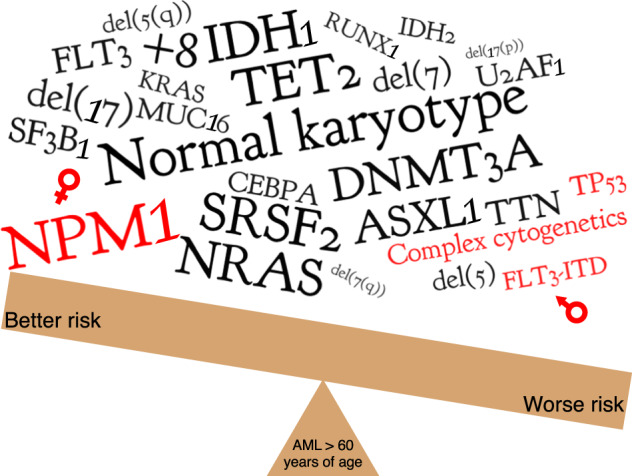

Dear Editor,

Acute Myeloid Leukemia (AML) is a heterogeneous hematological malignancy that most commonly presents in patients over the age of 60 (aged AML or aAML). aAML is associated with worse prognosis compared to younger adult AML patients [1]. The current molecular criteria considered for risk stratification (somatic mutations and cytogenetic abnormalities) were largely derived from molecular profiles of patients younger than 60 years of age [2]. Risk classifiers focused on aAML patients have been proposed [3, 4], but they only assessed selected gene mutations and/or did not include uniformly treated patients. While recent clinical trials with newly developed AML therapeutics [5, 6] might offer further insight into prognostication, comprehensive genomics data were not generated for further analyses. Thus, specific molecular determinants of clinical outcomes in aAML patients who are uniformly treated remain largely unknown.

To address this gap in knowledge, we performed whole exome sequencing (WES) on specimens collected from a clinically annotated aAML patient cohort enrolled in ECOG-ACRIN’s phase III clinical trial NCT00046930 [7] (Supplementary Table 1). These patients were uniformly managed and outcomes between experimental and placebo arms were not different, in trial results, and the patients from whom specimens were received (Supplementary Fig. 1). Centralized clinical and cytogenetics data were available. We assessed for recurrent somatic mutations in genes and cytogenetic events and performed association analyses to identify molecular events and clinical features predictive of clinical outcomes.

We first assessed the spectrum of somatic mutations in the patient samples in this study cohort. The cohort was characterized by 16 genes with recurrent oncogenic or likely oncogenic mutations (Supplementary Table 2), and each patient had an average of 3 oncogenic or likely oncogenic mutations (Fig. 1A, B). Most of the variants detected in these patients were variants of unknown significance (Supplementary Fig. 2). A subset of mutations were orthogonally validated using a custom targeted amplicon panel (Supplementary Fig. 3). This mutation rate was higher than that reported in patients younger than 60 (younger patients) in the BEAT AML [8] study cohort (validation cohort I; Wilcoxon test *P* = 1.44 × 10^–29^). We also observed a similarly increased mutation count in aAML patients compared to younger patients within validation cohort I (Wilcoxon test *P* = 9.48 × 10^−4^). This mutational increase might be due to age-related mutational processes where pre-leukemic cell clones would accumulate mutations prior to transformation into leukemic cells [9]. Consistently, we observed a significant enrichment of mutations in some known clonal hematopoiesis genes (*ASXL1, TET2, SRSF2,* and *U2AF1*) [9] (adjusted Fisher exact test *P* = 2.32 × 10^−3^, 3.73 × 10^−2^, 3.07 × 10^−3^, and 1.17 × 10^−4^; Fig. 1C; Supplementary Table 3). Clinical cytogenetics were available for a subset of patients from the study cohort (*n* = 166; Supplementary Table 4, Supplementary Fig. 4). When compared to younger patients in validation cohort I, we observed a significantly reduced frequency of MLL fusions and chromosome 16 inversions (adjusted *P* = 6.70 × 10^−3^ and 2.66 × 10^−2^ respectively; Fig. 1D; Supplementary Table 5).

**Fig. 1:**
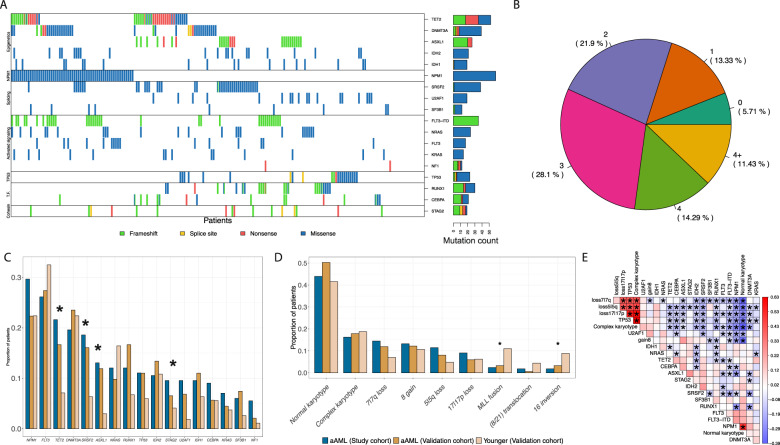
Somatic events landscape of the aged AML study cohort. **A** Co-mutation map for the cohort (*n* = 199). Each column is a gene and each row is a patient. Mutations of oncogenic or likely oncogenic significance were summarized by gene, with the exception of the FLT3-ITD mutation that was independently plotted. A cell is colored according to the type of mutation if a somatic mutation in the corresponding gene was found in the corresponding patient. Every gene that is mutated in at least 5% of the cohort (*n* = 9) is included. Colors: green is frameshift, yellow is splice site, red is nonsense, and blue is missense. Horizontal stacked bar graph represents the count summary for all mutation types per gene. **B** Percent of patients with oncogenic or likely oncogenic somatic mutations in the study cohort. **C** Bar plot of recurrent somatic mutations’ frequencies in the study cohort (blue), aAML patients in validation cohort I (brown), and AML patients younger than 60 in validation cohort I (tan). * is adjusted *p*-value < 0.05 from a Fisher’s exact test. **D** Bar plot of recurrent cytogenetic event frequencies in the study cohort (blue), aAML patients in validation cohort I (brown), and AML patients younger than 60 in validation cohort I (tan). * is adjusted *p*-value < 0.05 from a Fisher exact test. **E** Co-occurrence plot of the most common somatic events in the aAML study cohort. Mutations were summarized by gene, with the exception of FLT3-ITD that was independently plotted, and each cytogenetic event was summarized at the chromosomal level, with the exception of normal and complex karyotypes. Every event that is present in >5% of the cohort with available data (i.e., *n* > 9) is represented. Each cell represents the correlation between two events as measured by Pearson’s R with blue corresponding to mutually exclusive and red corresponding to co-occurring events. Asterisks indicate statistical significance (DISCOVER FDR < 0.05, see “Methods” for details). Pearson’s r = Pearson correlation coefficient (*r*).

When considering genes mutated in at least 5% of the patients independently (*n* = 199; Supplementary Fig. 5, Supplementary Tables 2 and 6) or with cytogenetics data (*n* = 166 patients; Fig. 1E; Supplementary Table 7), we found comparable patterns as to what has been reported for age unselected cohorts, including in validation cohort I [8]. A notable difference was mutual exclusivity between mutations in *NPM1* and *U2AF1* (FDR 2.96 × 10^−5^; previously reported in a retrospective analysis of other age-unselected cohorts [10]).

We next aimed to identify features (somatic and clinical features > 5%; *n* = 26; Supplementary Table 8) that were associated with overall survival (OS) in the study cohort. Complete molecular and outcomes data was available for 158 patients. Univariable analysis identified 13 distinct features that are associated with OS (*P* < 0.15; Supplementary Table 9). These 13 features were subsequently tested in recursive partitioning to identify patient subgroups with distinct outcomes. The terminal nodes of the model created 6 groups (G1–G6; Fig. 2A, B) based on five variables: Complex karyotype, mutations in *TP53*, *FLT3-*Internal Tandem Duplications (ITD), mutations in *NPM1*, and sex. We validated our findings in an independent aAML cohort (validation cohort II; Supplementary Tables 10A, B; Supplementary Fig. 6). Additionally, the six group classifier proved to be a better predictor of overall survival than ELN 2017 in the study cohort (ELN CPE = 0.624 and for the 6 group classifier CPE = 0.659 Supplementary Fig. 7A, B).

**Fig. 2:**
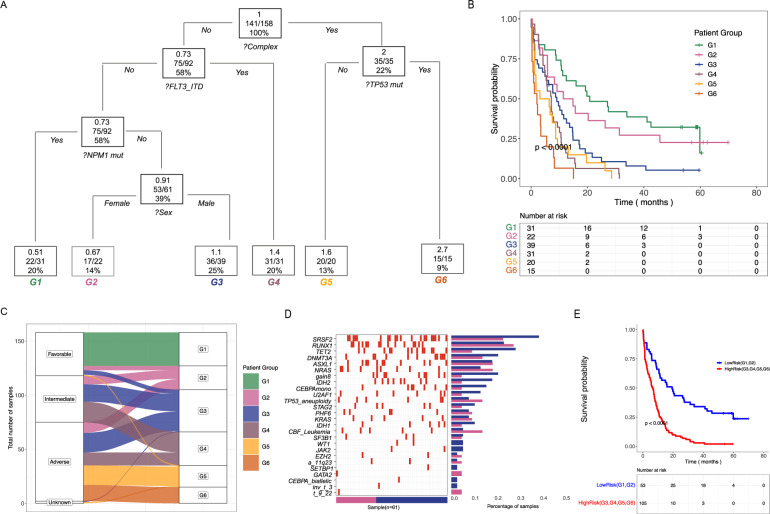
Integrative classifier identifies sex-associated outcomes. **A** Decision tree from the recursive partitioning analysis identified six distinct prognostic subgroups (G1–G6). Molecular characteristics of the group are defined as: G1 - Patients with non complex cytogenetics lacking FLT3-ITD mutations with mutations identified in *NPM1*; G2 - Female patients with non complex cytogenetics lacking FLT3-ITD and *NPM1* mutations; G3 - Male patients with non complex cytogenetics lacking FLT3-ITD mutations and *NPM1* mutations; G4 - Patients with non complex cytogenetics and FLT3-ITD mutations; G5 - Patients with complex cytogenetics and lacking *TP53* mutations; G6 - Patients with complex cytogenetics and mutations in *TP53*. Top number in each box is the hazard ratio, the middle ratio is the number of deaths/total number of patients in the tree branch considered, and the bottom number is the percent of the total number of patients in each tree branch. **B** Kaplan–Meier curves representing the survival probabilities in each of the six prognostic groups in the study cohort. The *p*-value was calculated using the log-rank test. **C** Proportional distributions of ELN categories (1 = favorable, 2 = intermediate, 3 = adverse, 4 = unknown) in the six prognostic subgroups (G1–G6). **D** Comparison of the frequencies of somatic mutations and cytogenetic events between the G2 and G3 prognostic groups. The plot on the left is a heatmap of the mutations in each gene per patient (red: mutated; white: wild type). The bar plot shows the percentage of samples with mutations in the gene of interest. **E** Kaplan–Meier curves comparing high (G3–G6) and low (G1, G2) risk groups in the study cohort. *P*-values were calculated using log-rank tests.

Our recursive partitioning analysis identified a novel group of patients solely consisting of females lacking complex cytogenetics, *NPM1* mutations and *FLT3*-ITDs (G2; Supplementary Fig. 8). The survival probability of G2 (0.66) was not different from that of a known non-M3 good risk AML patients harboring *NPM1* mutations [2] (OS probability 0.5; G1 in Fig. 2A; Supplementary Fig. 9). The survival probability of G2 was significantly better than patient groups (G4–G6; Fig. 2A, B) characterized by features previously identified to associate with poor clinical outcomes [2]. G2 patients had a higher incidence of achievement of complete remission compared to patients in G3 (Chi squared test *P* = 0.042; Supplementary Figure 10A), consistent with superior response to treatment. Furthermore, the survival probability of G2 was better than that of a group of males with the same genetic background (G3; Fig. 2A, B; Supplementary Fig. 10B).

The novel better risk group identified (G2) re-classified most female patients in this group from poor or intermediate ELN 2017 risk to good risk classification (Fig. 2C). Both G2 and G3 harbored poor and good risk molecular features. Some trends were observed suggesting differences in the frequencies of molecular events between G2 and G3, however, they were not significantly different between the groups (proportional test; *P* > 0.05; Fig. 2D and Supplementary Table 11). Furthermore, G2 and G3 did not have different mutation burdens (Supplementary Fig. 11; Wilcoxon rank-sum test *P* = 0.099), which may be the result of the small numbers of patients identified in each group. Nonetheless, this lack of difference in mutation burden suggests the possibility that there was no difference in DNA damage repair or chemotherapy response mechanisms that could contribute to differences in disease biology associated with the distinct clinical outcomes observed [11].

AML is more prevalent in males at any age, and it has already been reported that female pediatric and young adult AML patients had a better prognosis than males from the same age range [12]. We assessed for the potential applicability of survival differences between G2 and G3 patient groups to all adult AML patients over the age of 18. We analyzed outcomes in an AML cohort of adults younger than 60 years of age enrolled in ECOG-ACRIN clinical trial NCT00049517 (Supplementary Table 12) [13]. Applying our risk classifier did not identify a significant survival difference between men and women without complex cytogenetics, *NPM1* mutations, and *FLT3*-ITDs (Supplementary Fig. 12) in younger patients. This finding suggests that the novel risk group classification is specifically relevant to aAML patients.

We created two risk groups by visually comparing the Kaplan–Meier curves of the 6 terminal nodes (Fig. 2B). The low risk group included the two subgroups G1 and G2 (hazard ratios < 1) and the high risk group included the other four subgroups (hazard ratios > 1). We validated this separation using validation cohort II. Using our risk stratification, low risk patients had significantly better OS in both cohorts (log-rank tests *P* < 0.001; Fig. 2E and Supplementary Fig. 13). Furthermore, the groups were better associated with OS than standard ELN 2017 classification in validation cohort II (CPE for ELN 0.594 and for the new classifier 0.615).

Sex differences have been recognized in cancer incidence and outcomes, and may be an important factor in personalized treatment approaches. Previous publications have reported that AML female patients have overall better outcomes than male AML patients [12], however, this difference was not identified in NCT00046930 [12] (Supplementary Fig. 14), and analyses did not integrate genomics data. To our knowledge, this is the first report of integrative analysis between clinical and molecular events in aAML patients that has identified a classification in which sex serves as a risk predictor. The novel risk group identified reclassified a sub-group of female patients into a good risk category (Fig. 2C), which has implications for treatment selection in these cases [2]. Similarly, univariate (Supplementary Table 13) followed by recursive partitioning analysis also identified sex as a classifying parameter for the achievement of complete remission (Supplementary Fig. 15) in the study cohort. Our findings may be specific to the reported cohorts and require a larger study for confirmation. Future studies could improve upon the proposed risk classifier identified further by incorporating gene expression data, functional features such as BH3 profiling given growing interest in targeting apoptotic pathways in leukemia [14], as well as other laboratory values (e.g., serum LDH, albumin, or extreme leukocytosis). Intensive combination chemotherapy remains an upfront treatment option for fit aAML patients (NCCN 2022 guidelines), however, since upfront treatment options for aAML patients are evolving [15], independent assessments of the applicability of this risk classifier to emerging therapeutic approaches, such as Venetoclax combinations [15], will be required. If confirmed, this new risk assessment approach could inform age-appropriate risk stratification when evaluating the role of intensive combination chemotherapy induction treatment for aAML patients.

## Supplementary information

Supplementary Information

Supplementary Tables

## Data Availability

De-identified patient-level clinical and molecular data from NCT00046930 will be deposited into the National Institutes of Health’s NCTN/NCORP Data Archive (https://nctn-data-archive.nci.nih.gov) 6 months after publication. De-identified patient-level next generation sequencing data files will be deposited into the National Cancer Institute’s Cancer Data Service Portal (https://datacommons.cancer.gov/repository/cancer-data-service) 6 months after publication. Clinical and molecular data from validation cohort II are available upon request from carroll2@mail.med.upenn.edu. This manuscript utilized data from Dataset NCT00049517-D2 from the NCTN/NCORP Data Archive of the National Cancer Institute’s (NCI’s) National Clinical Trials Network (NCTN). Data were originally collected from clinical trial NCT00049517 entitled “Combination Chemotherapy With or Without Monoclonal Antibody Therapy in Treating Patients With AML Leukemia”. All analyses and conclusions in this manuscript are the sole responsibility of the authors and do not necessarily reflect the opinions or views of the clinical trial investigators, the NCTN, the NCORP or the NCI. During the review process, requested data files were available upon request to editorial staff and reviewers through direct invitations from the corresponding author (fg5q@virginia.edu) to a confidential Box folder.
