# Correction: Integrative analysis identifies an older female-linked AML patient group with better risk in ECOG-ACRIN Cancer Research Group’s clinical trial E3999

**DOI:** 10.1038/s41408-023-00862-2

**Published:** 2023-07-06

**Authors:** Franck Rapaport, Kenneth Seier, Yaseswini Neelamraju, Duane Hassane, Timour Baslan, Daniel T. Gildea, Samuel Haddox, Tak Lee, H. Moses Murdock, Caroline Sheridan, Alexis Thurmond, Ling Wang, Martin Carroll, Larry D. Cripe, Hugo Fernandez, Christopher E. Mason, Elisabeth Paietta, Gail J. Roboz, Zhuoxin Sun, Martin S. Tallman, Yanming Zhang, Mithat Gönen, Ross Levine, Ari M. Melnick, Maria Kleppe, Francine E. Garrett-Bakelman

**Affiliations:** 1 Human Oncology and Pathogenesis Program, Molecular Cancer Medicine Service, Memorial Sloan Kettering Cancer Center, New York, NY USA; 2 Center for Clinical and Translational Science, The Rockefeller University, New York, NY USA; 3 Department of Epidemiology and Biostatistics, Memorial Sloan Kettering Cancer Center, New York, NY USA; 4 Department of Biochemistry and Molecular Genetics, University of Virginia, Charlottesville, VA USA; 5 Division of Hematology and Medical Oncology, Department of Medicine, Weill Cornell Medicine, New York, NY USA; 6 Cancer Biology and Genetics Program, Sloan Kettering Institute, Memorial Sloan Kettering Cancer Center, New York, NY USA; 7 Division of Hematology and Oncology, University of Pennsylvania Perelman School of Medicine, Philadelphia, PA USA; 8 Simon Cancer Center, Indiana University, Indianapolis, IN USA; 9 Department of Malignant Hematology & Cellular Therapy, Moffitt Cancer Center, Tampa, FL USA; 10 Department of Physiology and Biophysics, Weill Cornell Medicine, New York, NY USA; 11 Institute for Computational Biomedicine, Weill Cornell Medicine, New York, NY USA; 12 The WorldQuant Initiative for Quantitative Prediction, Weill Cornell Medicine, New York, USA; 13 Montefiore Medical Center, Bronx, NY USA; 14 Weill Cornell Medicine and The New York Presbyterian Hospital, New York, NY USA; 15 Dana-Farber Cancer Institute, Boston, MA USA; 16 Memorial Sloan Kettering Cancer Center, New York, NY USA; 17 Department of Pathology, Memorial Sloan Kettering Cancer Center, New York, NY USA; 18 Department of Medicine, University of Virginia, Charlottesville, VA USA; 19 University of Virginia Cancer Center, Charlottesville, VA USA

**Keywords:** Cancer genetics, Cytogenetics, Cancer genetics

Correction to: *Blood Cancer Journal* 10.1038/s41408-022-00736-z, published online 23 September 2022

Following the publication of this article, the authors noted an error in sample reporting. Eleven specimens included in the original data were follow up specimens taken from patients after initial therapy, and not diagnostic specimens sampled prior to initial therapy.

To correct for this error, and to accurately report on findings in pre-therapy samples which have prognostic value, data from the 11 samples have now been excluded and the data re-analyzed.

Corrections and unchanged results after reanalysis are detailed as follows:

1. The overall number of genes with recurrent oncogenic and likely oncogenic mutations in the study cohort was unchanged (Fig. 1A, B, Supplementary Fig. 2).
2. The comparison to BEAT AML data and the findings of a subset of mutations being enriched in the older AML patients remains unchanged (Fig. 1C, D).
3. Cytogenetics data was not originally available for the specimens excluded, thus these analyses and published results are unchanged.
4. Conclusions from the somatic event co-occurrence analyses were unchanged (Fig. 1E, Supplementary Fig. 5).
5. Due to the smaller sample size, the threshold for inclusion in the regression tree analysis for association with overall survival was increased to keep the balance between false positive and false negative findings. After re-analysis, sex remains a classifying parameter for overall survival and the somatic events that contribute to the final model are unchanged (Fig. 2A, B).
6. Due to the smaller sample size, the assessment for higher frequency of achieving complete remission between G2 and G3 patient groups was changed from a fisher exact test to a chi-square test. With sufficient numbers in the 2 x 2 table (more than five in each cell) a chi-square test provides Type I control at the nominal level while supplying more power. We found a significant difference in achievement of complete remission between the groups as reported originally; the two groups also had a difference in overall survival, however, this difference was no longer significant (Supplementary Fig. 10; text lines 118–120).
7. The finding that the novel risk group reclassified most patients in the group from poor or intermediate ELN2017 to better risk was unchanged (Fig. 2C).
8. The findings when comparing the patterns of mutations in G2 and G3 risk groups were unchanged (Fig. 2D and Supplementary Fig. 11).
9. The finding of a better and worse risk group in the older AML patients remains unchanged (Fig. 2E).
10. Our findings regarding sex being a classifying parameter for achievement of complete remission in the study cohort remains unchanged (Supplementary Fig. 15).

To reflect the change in specimen count included, all figure panels listed above and associated text in the main paper, Supplementary Information and Supplementary Tables have been updated.

Fig. 1
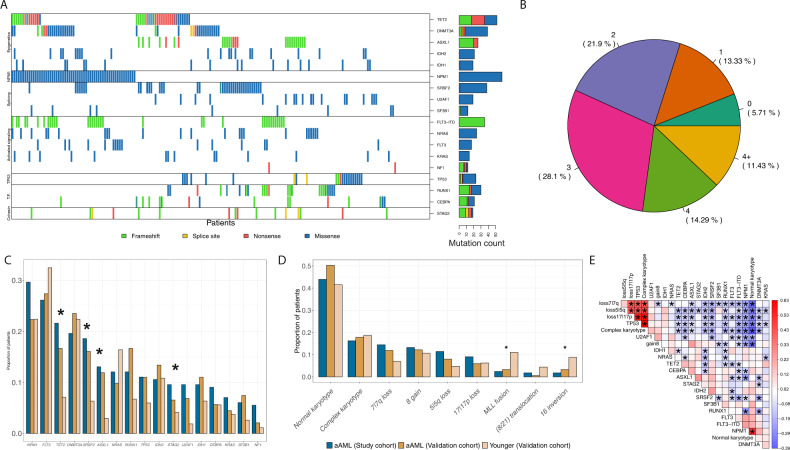


Fig. 2
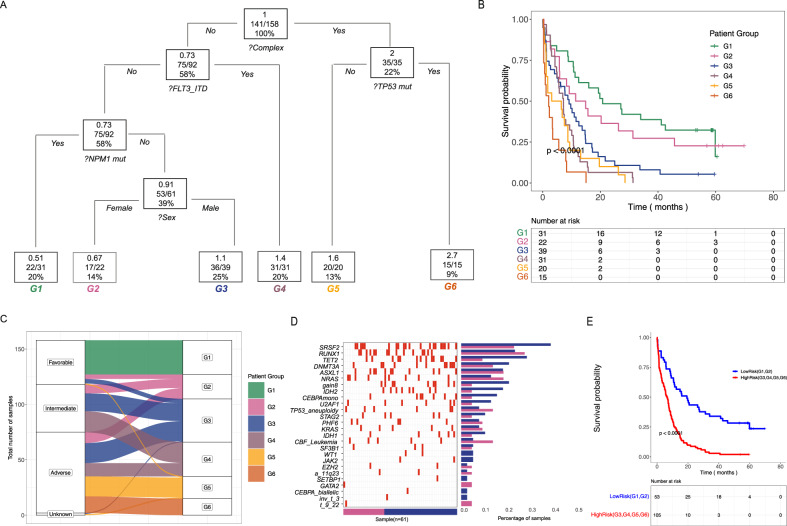


The original article has been corrected.

## Supplementary information

Supplementary Information

Supplementary Tables

