# Supplementary material for: Correction: Integrative analysis identifies an older female-linked AML patient group with better risk in ECOG-ACRIN Cancer Research Group’s clinical trial E3999
Source: Blood Cancer J. 2023 Jul 6;13(1):103. doi: 10.1038/s41408-023-00862-2 (PMC10322919; doi:10.1038/s41408-023-00862-2)
Supplement: Supplementary file 1 — Supplementary Information [file 41408_2023_862_MOESM1_ESM.pdf]

Integrative analysis identifies an older female-linked AML patient group with better risk in ECOG-ACRIN Cancer Research Group's clinical trial E3999

Supplementary Information

Table of Contents

Supplementary materials and methods 2
Supplementary figures and legends 8
Supplementary tables and legends 23
References 36

Supplementary materials and methods

Patient samples' collection and processing

All patients enrolled in ECOG-ACRIN clinical trial NCT00046930 (study cohort)[1] signed informed consent according to the declaration of Helsinki for collection and use of sample materials in research protocols. Study protocols were approved by the Institutional Review Boards at all participating institutions. Information on treatments received were reported in Cripe et al.[1] and will be included in metadata submitted to the NCTN. The trial randomized patients between combination intensive chemotherapy alone (Daunorubicin and Cytarabine) and addition of Zosuquidar, a P-glycoprotein inhibitor, or a placebo. Information about treatments received after the clinical trial (including stem cell transplantation) was not available. Since there was no survival benefit for Zosuquidar both in the original cohort[1] and the study cohort (Supplementary Figure 1), specimens from both arms were considered as a single group for analysis.

Study specimens were cells from diagnostic peripheral blood (PB) and bone marrow (BM) collections performed by ECOG-ACRIN. Mononuclear cells were isolated centrally on the day after collection and viably frozen in a 90:10% mixed solution of heat inactivated fetal bovine serum: dimethyl sulfoxide. De-identified specimens (n=239) were then provided to the study investigators. Samples were thawed and depleted of lymphocytes (CD3+ and CD19+ cells) using magnetic beads (Miltenyi Biotec, order No. 130-050-101 and 130-050-301 respectively) using the depleteS program on an autoMACS Pro Separator (Miltenyi Biotec, Bergisch Gladbach, Germany) to enrich for disease cells. T-lymphocytes were then isolated for patient-matched germline controls using flow sorting for viable CD3+ on a FACSAria II cell sorter (BD Biosciences, San Jose, CA; anti-human CD3 antibody-PE-CY7 from Biolegend, clone SK7, Order No. 344816 and anti-human CD19 antibody-APC from Biolegend, clone HIB19, Order No. 302211). Validation for each antibody is provided on the manufacturer's website (http://www.biolegend.com/).

DNA was isolated from the disease and lymphoid cell fractions using Qiagen's Gentra Puregene Kit (Cat. No. 158667) per manufacturer's recommendation. Quality control was performed using standard agarose gel visualization and DNA was quantified using ThermoScientific's Qubit 2.0 Fluorometer and Qubit™dsDNA HS Assay Kit (ThermoScientific, Cat. No. Q32854).

Cytogenetic studies

Cytogenetic analyses of pretreatment BM and/or PB samples were reviewed centrally. For the normal karyotype determinations, at least 20 metaphases from pretreatment specimens subjected to short-term (24–48 hours) cultures had to have been analyzed without any clonal abnormalities identified.

Exome capture protocol and sequencing

There was sufficient material extracted from myeloid enriched and T-lymphocyte cell populations in n = 205 to perform exome capture. Next generation sequencing libraries were prepared from 50ng input DNA using Agilent SureSelectXT V4 51mb Exome kit per manufacturer's recommendations. Sequencing was performed on an Illumina HiSeq 2500 using 125bp paired-end

sequencing to a mean depth per target area of 94.6X (for tumors) and 91.6X (for matched germline samples). Sequencing statistics summary is included in Supplementary Table 14.

Data alignment and somatic event annotation

The reads were aligned to the human genome (UCSC build hg19) using the Burrows-Wheeler Aligner with maximal exact matches[2]. We used the Cancer Genome Project pipeline (https://github.com/cancerit) and compared the tumor samples to their matched germline samples following the pipeline recommendations. Snpeff[3] was used to annotate variants with their functional consequences. We filtered out variants that were present in any of the population groups from the ExAC database[4] with a minor allele frequency of more than 1%. We annotated variants as "unknown significance", "likely oncogenic" or "oncogenic" according to previously outlined criteria[5]. Six samples were excluded due to poor quality.

***NPM1* indel detection**

NPM1 indels in exon 12 were detected using fragment analysis. Fragment analysis was performed as previously described[6]. Briefly, 10 ng of genomic DNA was amplified in 25 µl reactions containing 1X KAPA HiFi HotStart Ready Mix (Kapa Biosystems catalog number KK2602-07958935001), 0.3 mM NPM-F primer (6-FAM-5'-GATGTCTATGAAGTGTTGTGGTTCC-3'), 0.3 mM NPM-R primer (5'-GGACAGCCAGATATCAACTG-3'), and nuclease-free water. PCR was performed using an Eppendorf MasterCycler (Hamburg, Germany) with the following cycling conditions: initial denaturation at 95 °C for 2 minutes; 26 cycles of 95 °C for 20 seconds, 60 °C for 20 seconds, 72 °C for 20 seconds; final extension at 72 °C for 2 minutes. 6-FAM-labeled amplicons were isolated using a 1.8X ratio of Agencourt AMPure XP beads (Beckman Coulter) and eluted in 20 µl EB buffer. 10 µl of the eluate was submitted to GENEWIZ for fragment analysis. Fragment analysis results were analyzed with Peak Scanner Software 2 (Applied Biosystems).

***IDH1* mutation detection**

IDH1 R132 mutations were assessed for using targeted polymerase chain reaction (PCR) followed by Sanger sequencing as previously described[7]. Briefly, a 420 base-pair region of *IDH1* coding for R132 was amplified from 2-25ng of purified genomic DNA using Q5 Hot Start High-Fidelity 2x Master Mix (New England BioLabs, IPSWICH, MA) and the following primers: IDH1-F GTACTCAGAGCCTTCGCTTTCTGC, IDH1-R GCCAACATGACTTACTTGATCCCC. PCR was performed using an Eppendorf MasterCycler (Hamburg, Germany) with the following cycling conditions: initial denaturation at 95°C for 2 minute; 35 cycles of 95°C for 15 seconds, 59°C for 15 seconds, and 72°C for 30 seconds; final extension at 72°C for 2 minutes. Specific PCR products were confirmed by melt-curve analysis and agarose gel electrophoresis before submitting to GENEWIZ for Sanger sequencing (South Plainfield, NJ). Trace files were aligned with human genome assembly GRCh37/Hg19 to assess base pairs 209113112 and 209113113 for substitute mutations (NCBI BLAST: https://blast.ncbi.nlm.nih.gov/Blast.cgi).

FLT3-ITD detection

The presence of an Internal Tandem Duplication (ITD) in the *FLT3* gene between exons 14 and 15 was assessed through standard PCR as previously described[8]. Briefly, we used 10-50 ng of genomic DNA as template in a 10 µl total volume reaction and 2% agarose gel electrophoresis. PCR was carried-out with the following primers and cycling conditions: 11F-GCAATTTAGGTATGAAAGCCAGC, 12R-CTTTCAGCATTTTGACGGCAACC, initial denaturation at 94°C for 3 min followed by 35 cycles of 94°C for 30 sec, 56°C for 1 min, and 72°C for 2 min. A 329 bp wild type (WT) product was amplified by these two primers with larger bands indicating occurrence of FLT3-ITD.

The presence of an ITD in FLT3 mRNA between exons 14 and 15 was validated in a subset of specimens through qualitative RT-PCR using 40-60 ng of total RNA as template for reverse transcription. Reverse Transcription was carried out at 42˚C for 1.5 hours with M-MuLV Reverse Transcriptase (NEB Cat# M0253L Ipswich, MA), and a 3:1 mixture of random hexamers (ThermoFisher Cat# SO142 Waltham, MA), to Anchored Oligo-dT, (IDT Coralville, IA) in a 20 µl total volume reaction. Subsequently, cDNA was diluted 6-fold and 4µl of diluted cDNA was used in each 10µl PCR reaction with the following primers, R5-tgtcgagcagtactctaaaca and R6-atcctagtaccttcccaaactc[9]. Amplification cycling included an initial denaturation at 94°C for 3 min followed by 40 cycles of 94°C for 30 sec, 56°C for 1 min, and 72°C for 2 min. A PCR product of 366 bp was generated from WT *FLT3* with larger bands indicating the presence of ITDs.

All PCR was carried-out using an Eppendorf MasterCycler (Hamburg, Germany) and Taq 2x Master Mix M0270L (New England BioLabs, IPSWICH, MA) according to the manufacturer's instructions.

Molecular functional annotation

Gene mutations and cytogenetic events were annotated to groups similar to previous reports[5,10–14] as follows: Core binding factor (CBF: t(8;21) (q22;q22.1) and inv(16)(p13.1q22)), loss 7/7q (monosomy chromosome 7 or 7(q) abnormality), loss 17/17(p) (monosomy of chromosome 17 or 17(p) abnormality), loss 5/5(q) (monosomy of chromosome 5 or 5(q) abnormality), complex karyotype (three or more unrelated chromosome abnormalities in the absence of 1 of the WHO-designated recurring translocations or inversions), tumor suppressors (*PHF6, TP53*), Splicing (*SRSF2, U2AF1, SF3B1*), Epigenetics/DNA methylation (*DNMT3A, IDH1, IDH2, TET2, WT1,* and *ASXL1*), Activated signaling (*FLT3, NRAS, KRAS, CBL, JAK2, MPL* and *NF1*), Chromatin regulation (*ASXL1* and *EZH2*), Transcription factors (*RUNX1* and *CEBPA*), and Cohesin (*STAG2*) for analyses.

Validation targeted sequencing.

Validation of somatic mutations was performed using a custom targeted amplicon panel. The custom targeted amplicon panel was performed as previously described[15]: Briefly, 1. Library generation and amplification were performed using a low error rate Hi-Fi DNA polymerase according to the Kapa HyperPrep protocol (Kapa Biosystems). 2. Targeted sequencing using a panel of 22 recurrently mutated genes in hematological malignancies was performed using a

custom capture probe set targeting all exons (Integrated DNA Technologies; genes included: *ABL1, ASXL1, BCOR, BCORL1, CALR, CBFB, CEBPA, DNMT3A, FLT3, IDH1, IDH2, JAK2, KIT, KMT2A, KRAS, NPM1, NRAS, RARA, RUNX1, TET2, TP53, WT1*) per manufacturer's recommendations. 3. Following targeted enrichment performed per Nimblegen protocols, libraries were sequenced on an Illumina HiSeq 4000 using dual-indexed sample adapters (Integrated DNA Technologies) to a median coverage of 2000x. 4. Reads were trimmed of contaminating adapter sequences and low- 31 quality bases using Trimmomatic v0.32[16] (trimmed when median Illumina base quality score < 20 over 632 nt sliding window). Overlapping paired end reads were merged into a single long consensus read using AdapterRemoval v241 when at least 12 bp overlap was present. 5. The remaining high-quality reads were aligned to the 1000 Genomes Phase 2 human reference genome and decoy contigs (hs37d5) using BWA MEM[2]. Duplicate marking was performed using SamBlaster v0.1.21[17] and MarkDupsByStartEnd v0.2.1.6 (https://github.com/dariober/Java-cafe/tree/master/MarkDupsByStartEnd). Single nucleotide variants (SNVs) and insertions/deletions (indels) were detected using VarDictJava v1.4.6[18] in single sample mode with indel realignment. 7. Annotation of variants and their functional impact was performed using Variant Effect Predictor (VEP) v8547[19] and snpEff v4.1g.8[3]. To identify somatic variants, filtration based on population allele frequency data was applied so as to enrich somatic variants that were not likely inherited. To this end, variants were classified as probable somatic if exhibiting a dbSNP v142[20] or ExAC[4] adjusted population allele frequency <= 0.25% or a median VAF of 2.5%. Variants that occurred at a VAF of 5% were compared to the exome capture results.

Co-occurrence analyses.

Co-occurrence analyses of somatic events (mutations and cytogenetic events) was performed on events that occurred at a frequency of greater than 5% in the study cohort. We performed a similar analysis including every sample on genes mutated in more than 5% of the study cohort. For both of these analyses, we considered *FLT3*-ITD and other *FLT3* mutations as two separate events. The analysis was performed utilizing the pairwise.discover function from the Discrete Independence Statistic Controlling for Observations with Varying Event Rates[21] version 0.9.3 once with default parameters and once with the parameter alternative="greater". Findings in the study cohort were compared to results reported for validation cohort I[22].

Outcome association analyses.

Outcome association analyses for overall survival (OS; time from diagnosis to death or last follow up) were performed on the study cohort. To compare between groups, Fisher's exact test was used for categorical and Wilcoxon rank sum test was used for numerical variables. We assessed somatic mutations, clinical features and cytogenetic events with a minimum of 5% prevalence for association with OS. Prevalence was described by percentage and confidence interval. Cox proportional hazard models (SAS proc phreg) were used for assessment of features' association with OS. To achieve our goal of defining subgroups, we used recursive partitioning decision trees (R function rpart), rather than traditional multivariable analysis. The recursive partitioning analysis

included distinct variables (excluding grouped categories) significant at alpha = 0.15 or lower from the univariable analysis to identify subgroups associated with OS[23]. The subgroups from the terminal nodes of the decision tree were then categorized into low vs high risk groups based on Kaplan Meier curves. Log-rank tests were implemented for assessment of group differences. We used external data from validation cohort II to validate our new risk groups using Kaplan Meier curves and log rank test (SAS proc lifetest). The ability to predict OS of the new classifier was compared to the European LeukemiaNet (ELN) 2017 classification[24]. Since *FLT3*-ITD's were assessed qualitatively from standard PCR, low versus high variant allele frequencies could not be robustly determined. The presence of a *FLT3*-ITD classified a patient as intermediate risk if it co-occurred with a mutation in *NPM1*, and classified a patient as adverse risk if it occurred otherwise. Our classifier was compared to ELN's patient classification annotation using the concordance probability estimate[25] (CPE; R packages RMS and CPE). Analyses were performed with SAS version 9.4 (SAS institute Inc., Cary, NC) and R version 3.6.1. All tests were two-sided and $p<0.05$ was considered significant.

The secondary outcome was achievement of complete response after induction chemotherapy treatment (complete remission; CR). Univariable logistic regression was used for assessment of mutations' association with the achievement of CR (SAS proc genmod). We used recursive partitioning decision trees (R function rpart) that included variables significant at alpha = .1 from the univariable analysis to find important subgroups associated with achievement of CR[23]. Analyses were performed with SAS version 9.4 and R version 3.6.1. All tests were two-sided and $p<0.05$ was considered significant.

Adjustments for stem cell transplantation effects and minimal residual disease were not possible since documentation available from NCT00046930 did not include this information. Differences in the overall survival times between groups were plotted using Kaplan-Meier curves with log-rank tests utilizing survival (v 3.2-7) and survminer (v 0.4.9) packages in R (v 3.6.3).

Datasets

Validation cohort I

We obtained somatic mutation data from an independent cohort of 604 AML samples (Beat AML)[22], consisting of 336 samples from patients older than 60 years of age and 268 samples from patients younger than 60 years of age. We compared the mutational landscape of these two groups to the aAML cohort using an adjusted Fisher's exact test (R version 4.0.2 base functions fisher.test for the test and p.adjust for adjusting the P-values). Data is available in the supplementary materials of the original publication.

Validation cohort II

We obtained cytogenetic and somatic mutation data from an in house panel, and clinical information for 95 aAML patients diagnosed between 2001 and 2013 and treated with intensive induction combination chemotherapy at the University of Pennsylvania. Four of the patients were previously included in Rapaport et al.[26]. Clinically-annotated cytogenetics and somatic mutations in *NPM1* and *TP53* genes as well as *FLT3*-ITD status were available for 87 patients. One patient

was excluded due to being enrolled in the NCT00046930 clinical trial from which the study cohort specimens were obtained, three patients treated with clofarabine were excluded due to reported differences in outcome compared to intensive combination chemotherapy induction treatment[27], and two patients treated with low dose cytarabine without an anthracycline were excluded. The remaining 81 patients were treated with intensive combination chemotherapy induction treatment. The 81 patients were categorized into the six groups based on the annotations used in the study cohort. Data is available upon request from carroll2@mail.med.upenn.edu.

Outcome analysis in NCT00049517

We obtained the somatic mutation data and metadata (age, sex and treatment) from 497 patients younger than the age of 60 enrolled in the E1900 clinical trial (NCT00049517)[28,29]. The patients in this cohort considered in analyses underwent two different treatment regimens. Patients categorized in Cohort 1 (n = 176) received standard (low) dose Daunorubicin+Cytarabine with or without allotransplantation and Cohort 2 received (n = 158) high dose Daunorubicin+Cytarabine with or without allotransplantation. The cohorts showed a significant difference in their survival outcomes and hence were analyzed independently. Patients from each cohort was further assigned into our defined G2 and G3 groups using clinical, cytogenetics and molecular data available from the cohort (sex, complex karyotype, *FLT3*-ITD, *NPM1* and *TP53* mutations).

Supplementary figures and legends

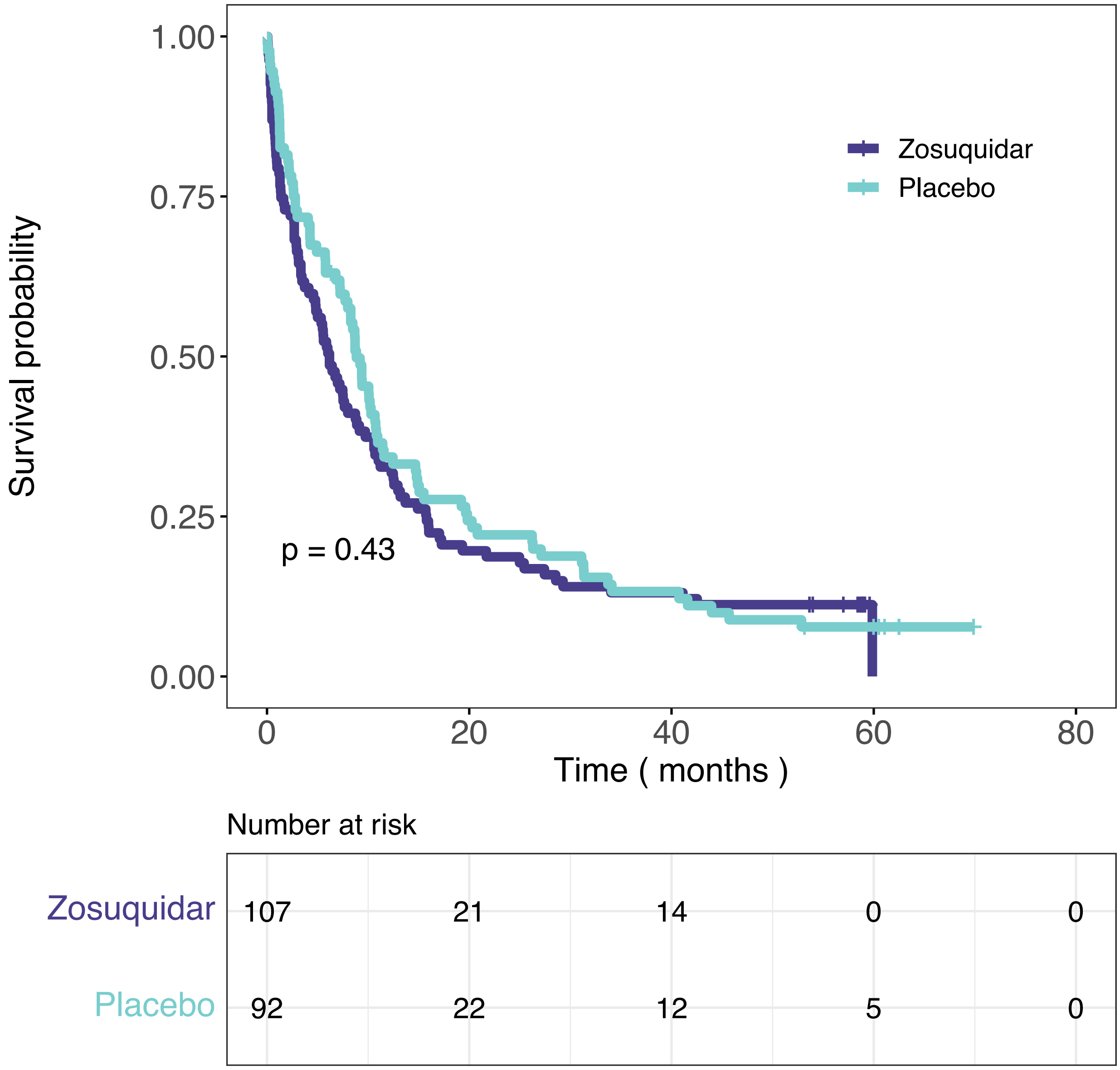

Supplementary Figure 1: Survival characteristics of study cohort. Kaplan Meier plot representing the survival probabilities of the experimental and placebo groups in the study cohort. p-value was calculated using the log-rank test.

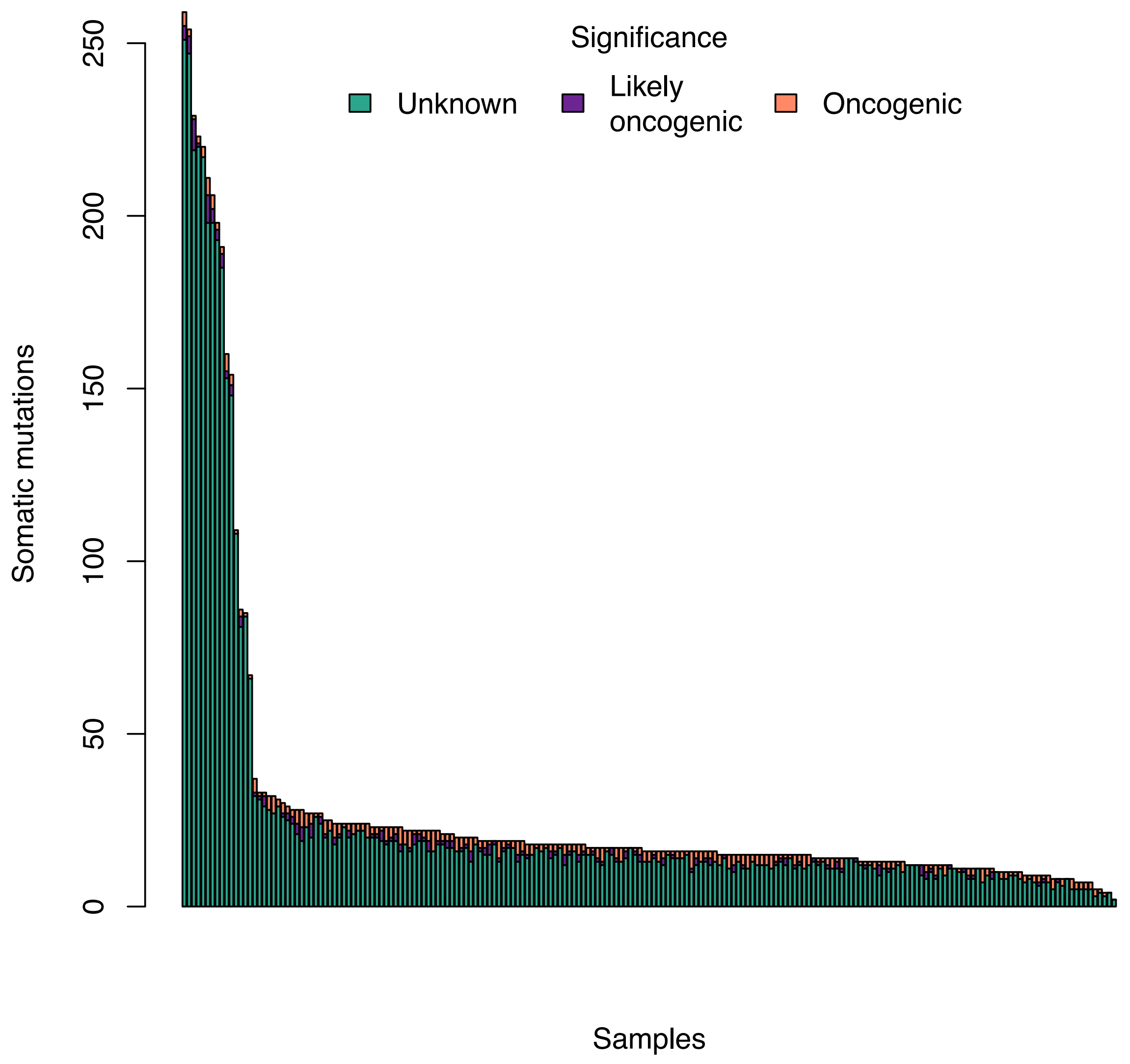

Supplementary Figure 2: Mutational burden in the study cohort. Number of somatic mutations (single nucleotide variants and short insertions/deletions) per patient, colored by the significance of the mutation (teal: unknown significance, purple: likely oncogenic, coral: oncogenic).

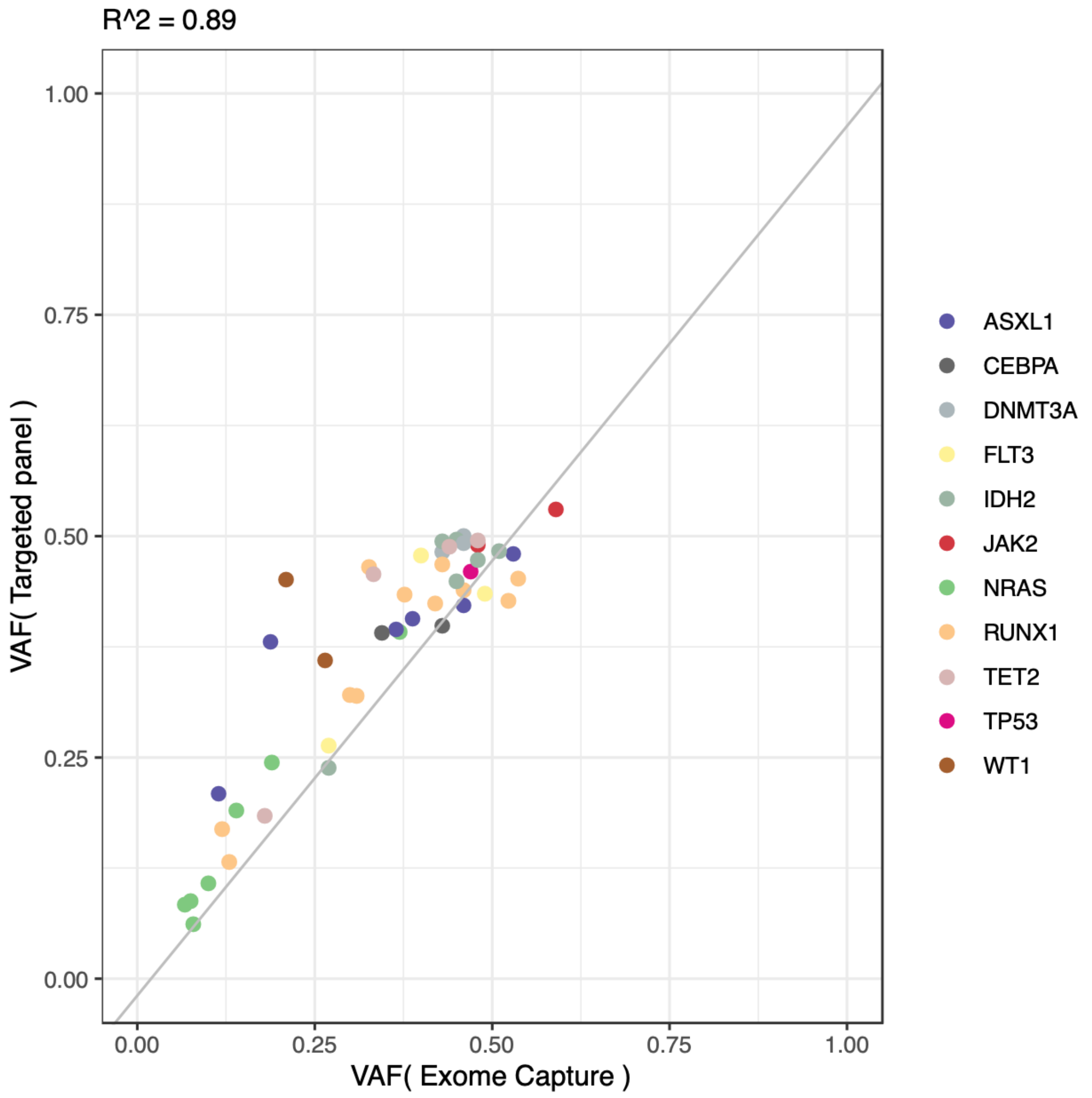

Supplementary Figure 3: Validation of somatic mutations using a custom targeted amplicon panel. Scatter plot comparing the variant allele fraction (VAF) from the WES assay and the targeted panel. Pearson correlation was used to assess the similarities in the VAFs identified in both approaches (R squared = 0.89).

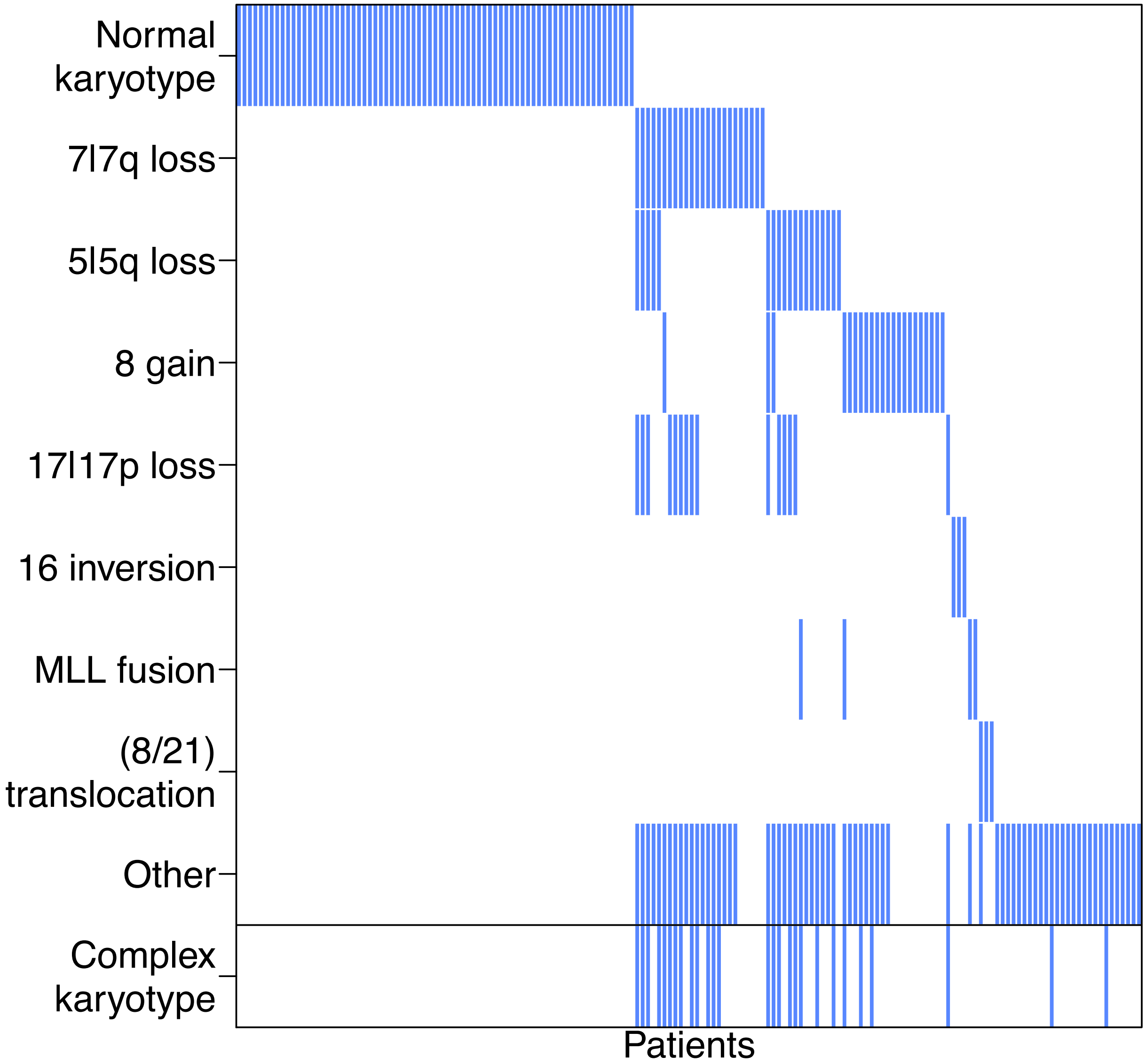

Supplementary Figure 4: Cytogenetic events in the study cohort. Distribution of the cytogenetic events in the study cohort (n = 166). Each row is an event, and each column a patient. Normal karyotypes are included. Cytogenetic events were summarized at the chromosomal level, with the exception of MLL fusions and t(8;21) translocations. Every event that was found in at least 3 patients is included. Complex karyotype was defined as 3 or more unrelated cytogenetic events.

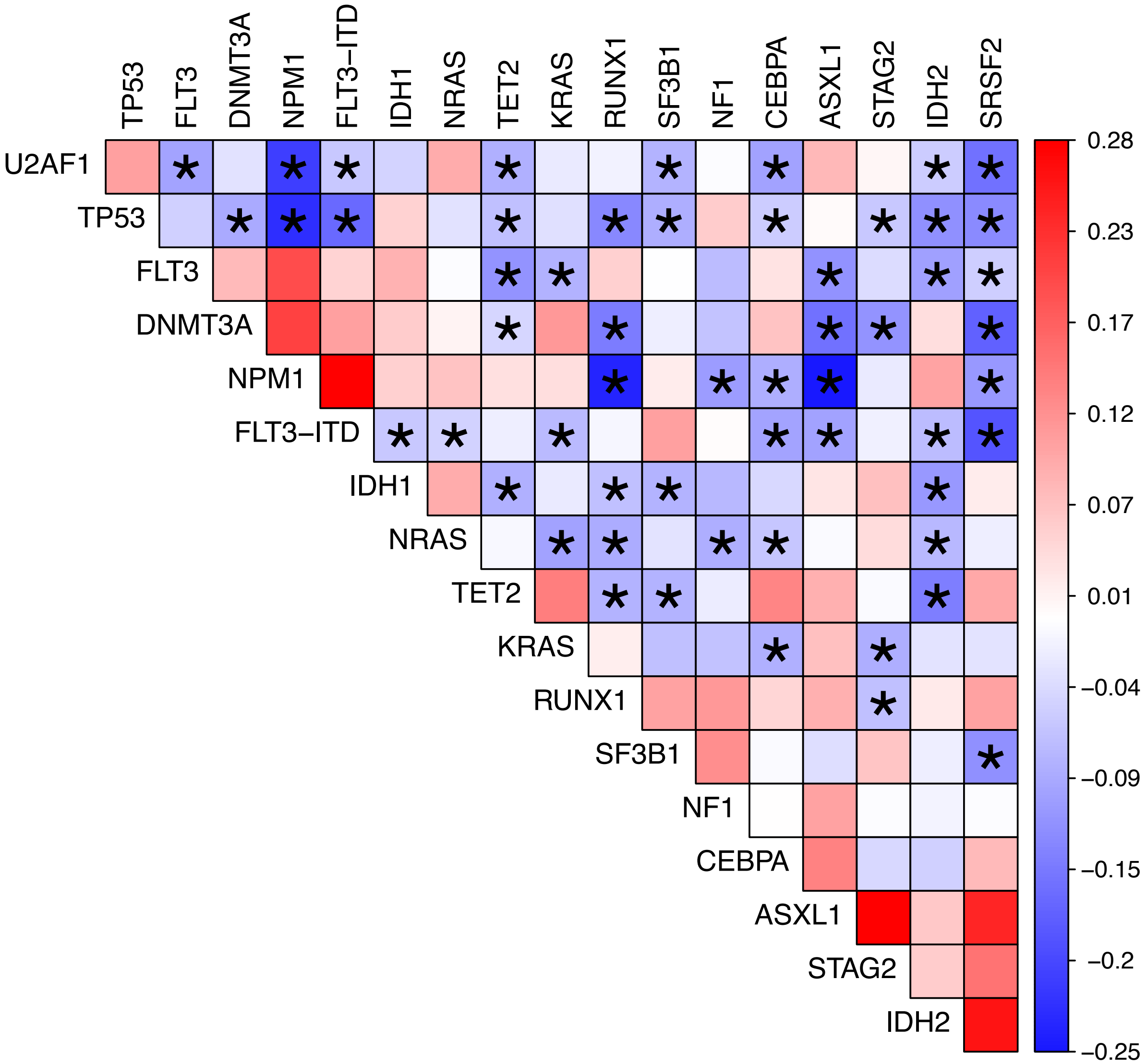

Supplementary Figure 5: Co-occurrence or exclusivity of the most common somatic mutations (single nucleotide variants and short insertions/deletions; n = 199). Mutations were summarized by gene, with the exception of *FLT3*-ITD that was independently plotted. Every gene that is mutated in >5% of the cohort (n > 9) is shown. Each cell represents the correlation between two events as measured by Pearson's R with blue corresponding to mutually exclusive and red corresponding to co-occurring events. Asterisks indicate statistical significance (DISCOVER FDR < 0.05, see Methods for details). Pearson's r = Pearson correlation coefficient (r).

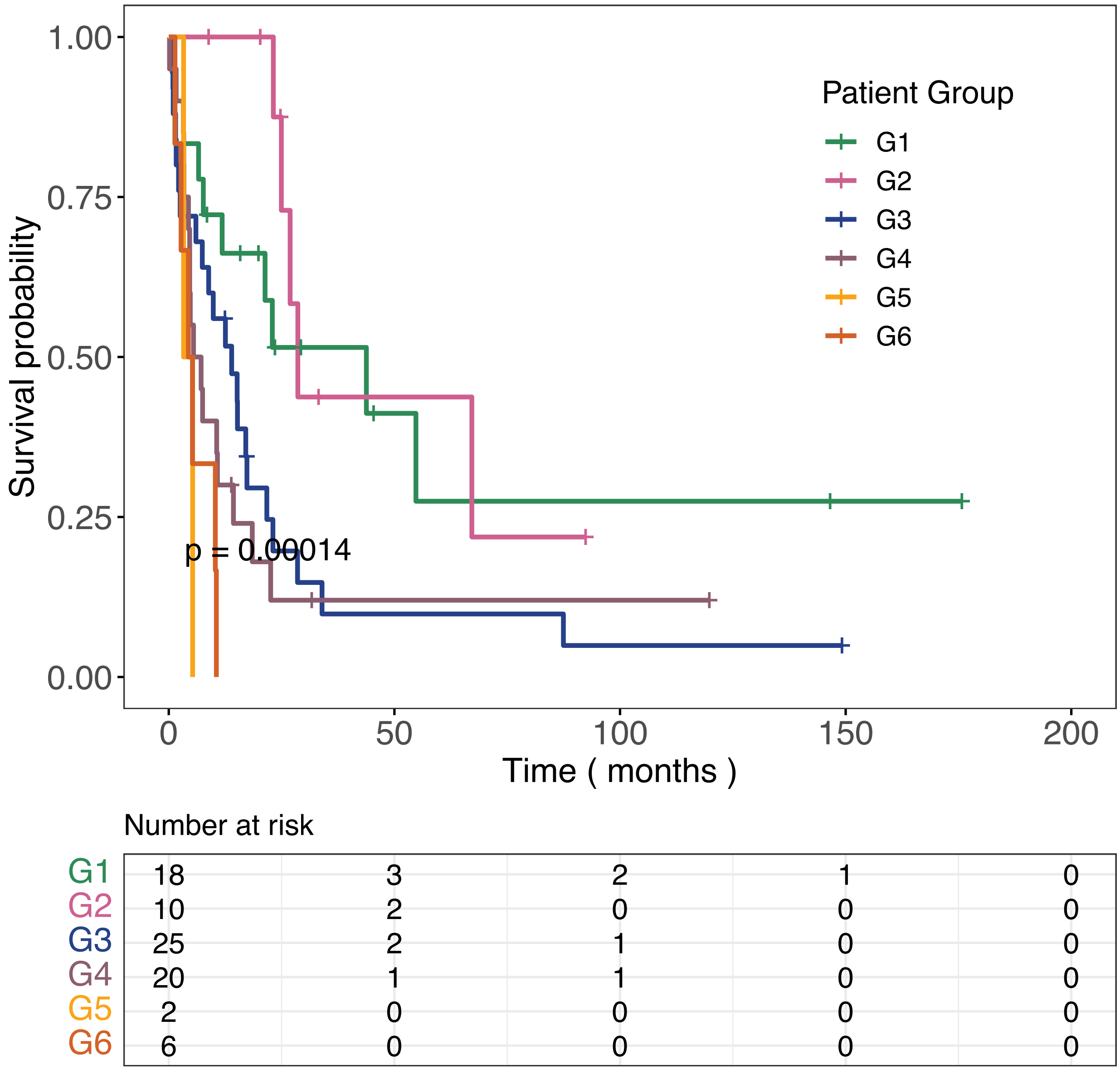

Supplementary Figure 6: Overall survival analysis in validation cohort II. Kaplan-Meier curves representing the survival probabilities in each of the six prognostic groups (identified in the recursive partitioning analysis performed in the study cohort) in validation cohort II. The p-value was calculated using the log-rank test.

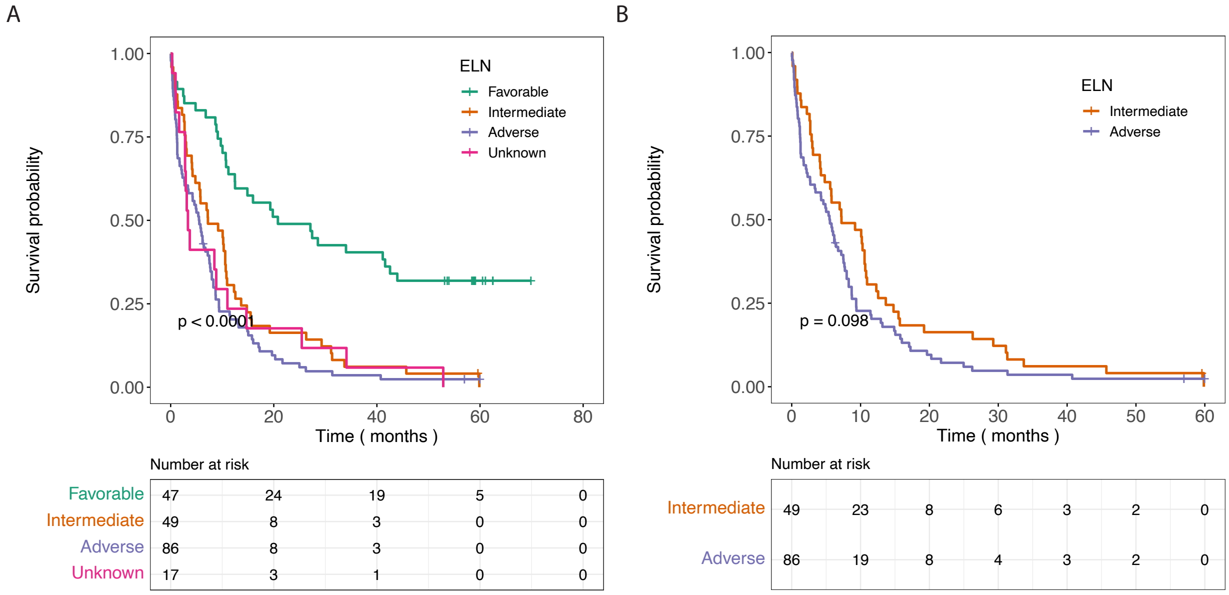

Supplementary Figure 7: ELN2017 survival characteristics of study cohort. A) Kaplan Meier plot representing the survival probabilities of ELN classified patient groups in the study cohort. B) Kaplan Meier plot representing the survival of ELN intermediate and adverse risk classified patients in the study cohort. p-value was calculated using the log-rank test.

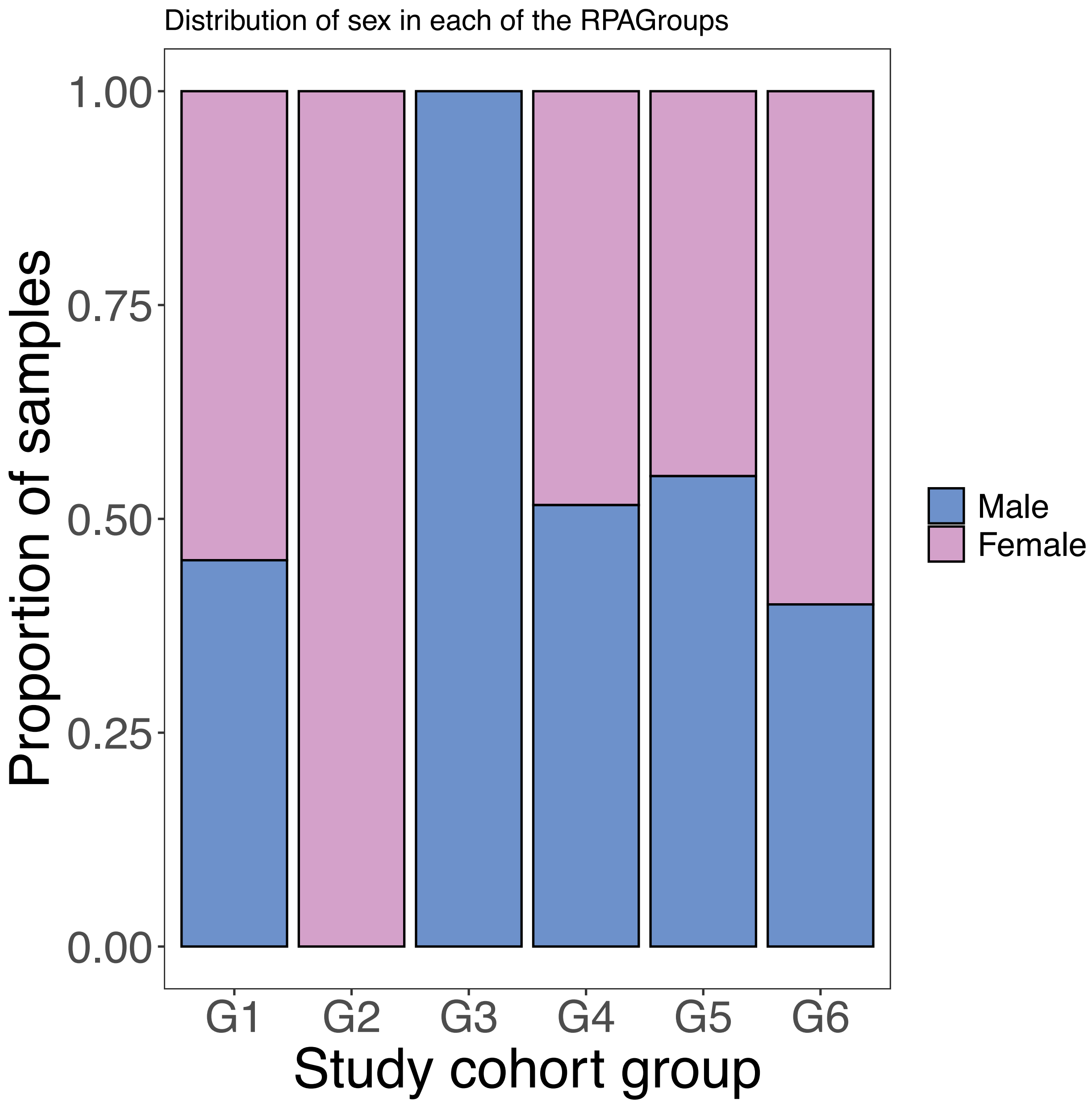

Supplementary Figure 8: Proportion of female and male sex patients in six prognostic groups. Stacked bar plots representing the proportion of females and males in each of the six prognostic groups identified in our study cohort.

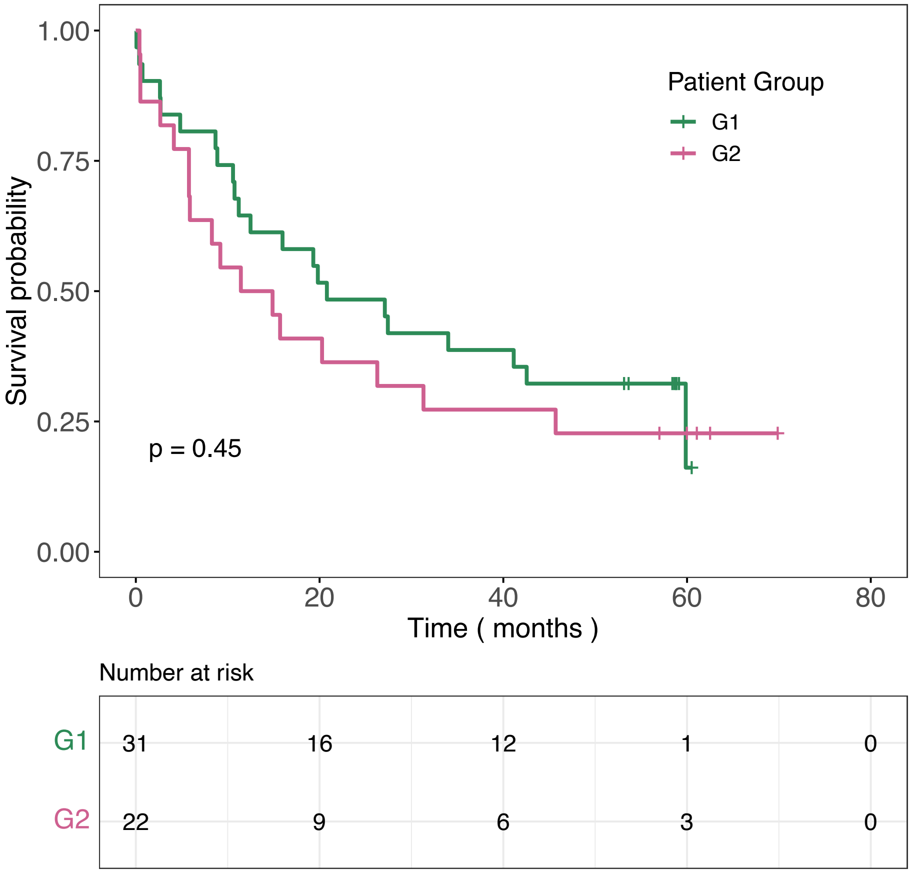

Supplementary Figure 9: Kaplan-Meier curves of study cohort groups. Kaplan-Meier curve comparing the survival probabilities between G2 and G1. P-value in the plot was calculated using the log-rank test.

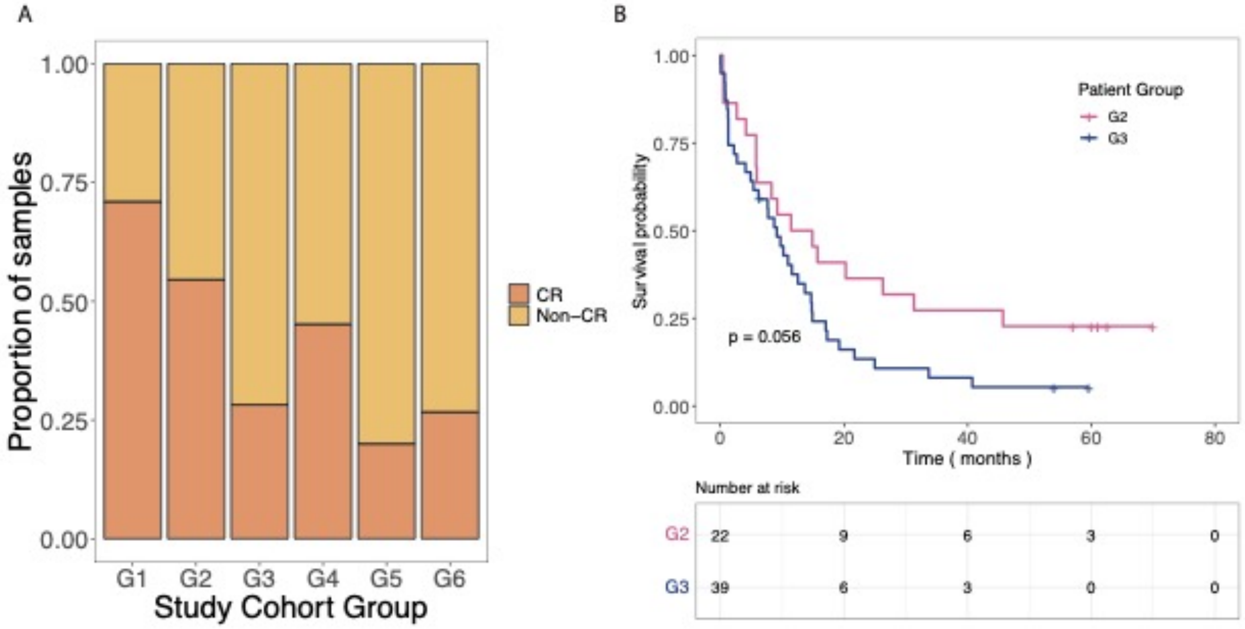

Supplementary Figure 10: Comparison of clinical outcomes between study groups G2 and G3. A) Stacked bar plot of patients that achieved CR (orange) or did not achieve CR (yellow) in each of the study cohort patient groups G1 – G6. Comparison tests were fisher's exact test (* indicates p = < 0.05). B) Kaplan-Meier curve comparing the survival probabilities between G2 and G3 patients. P-value in the plot was calculated using the log-rank test.

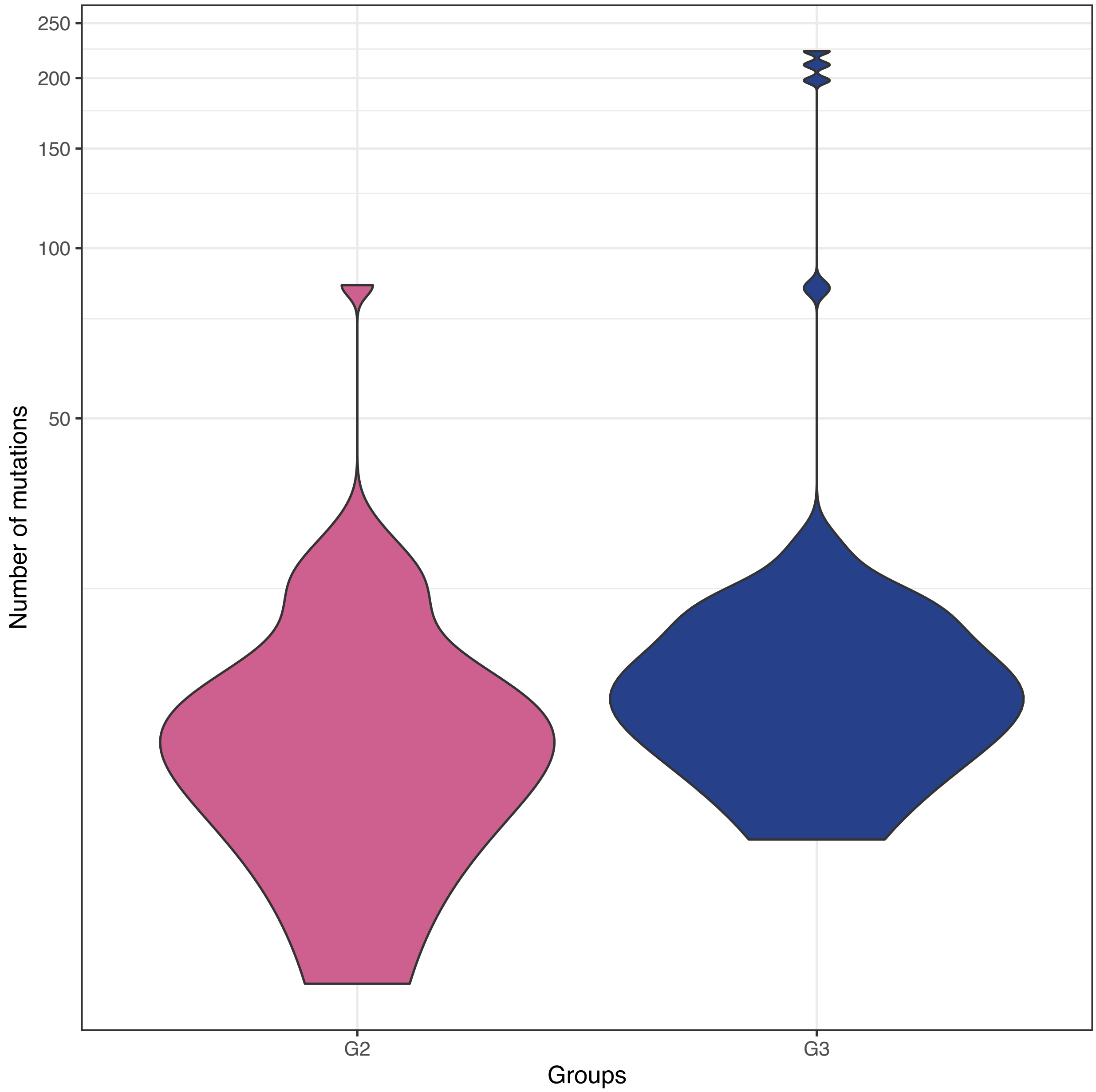

Supplementary Figure 11: Mutational burden comparisons between study cohort patient groups G2 and G3. Distribution of mutation burdens in G2 and G3 group study cohort patient specimens.

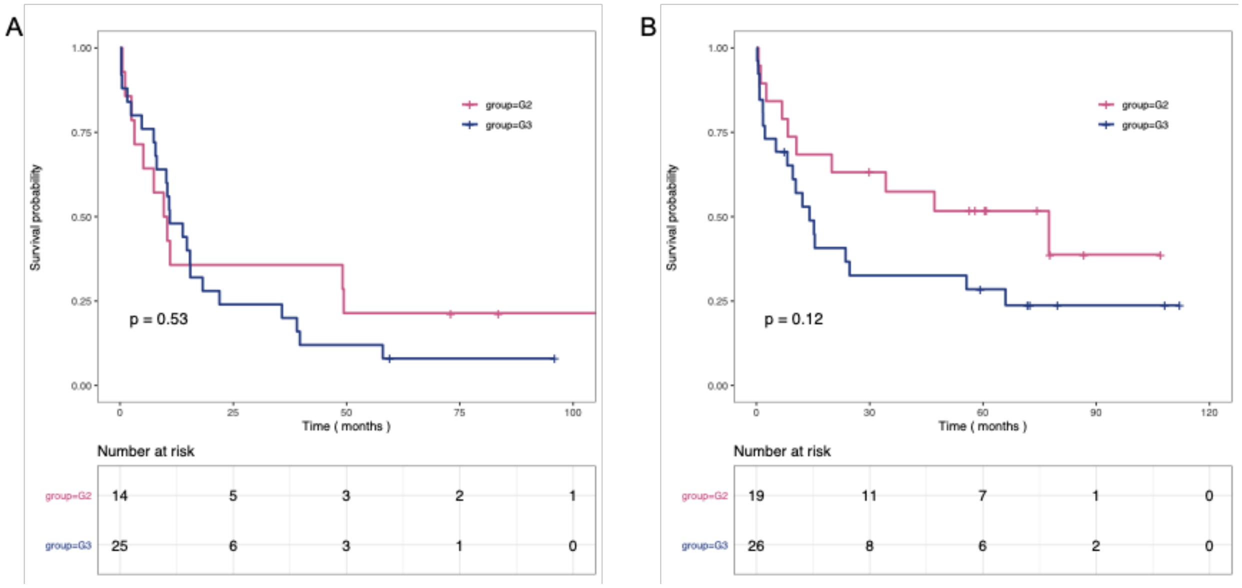

Supplementary Figure 12: Outcome analysis in an AML cohort younger than 60 years of age. A) Kaplan Meier curves comparing overall survival between G2 and G3 annotated patient groups in the low dose treatment cohort. B) Kaplan-Meier curves comparing overall survival between G2 and G3 annotated patient groups in the high dose treatment cohort . P-values in all the curves were calculated using the log-rank test.

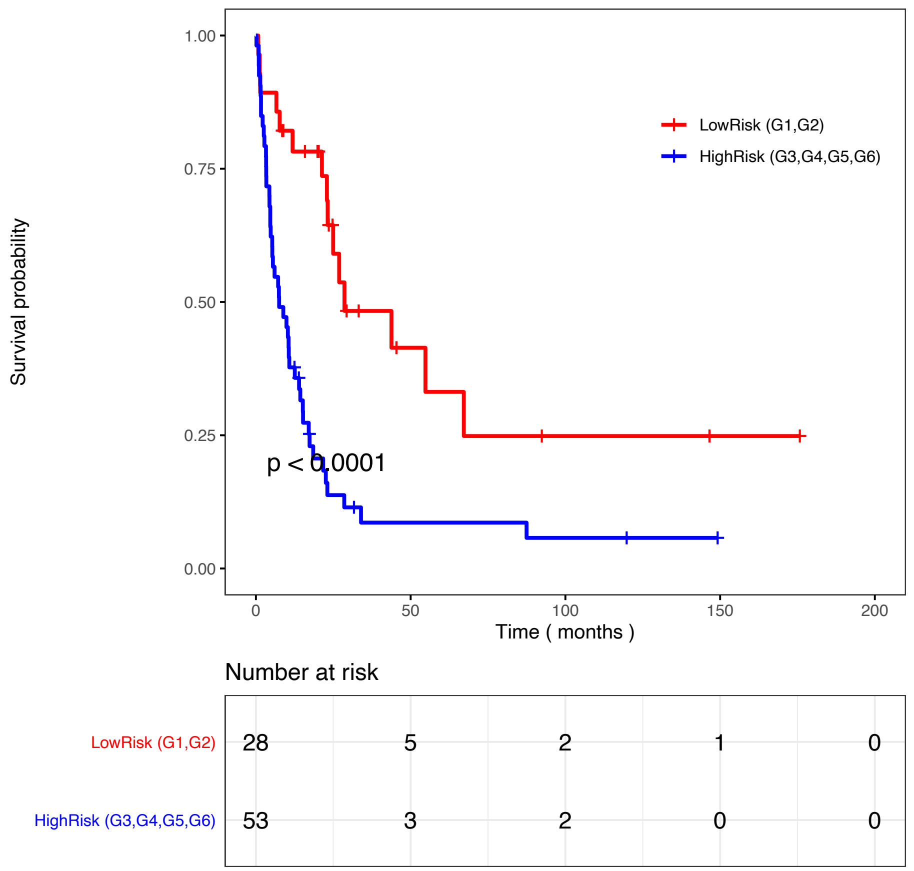

Supplementary Figure 13: Low and high risk classification result in validation cohort II. Kaplan-Meier curves comparing high (G3-G6) vs low (G1,G2) risk groups in validation cohort II. P-value was calculated using a log-rank test.

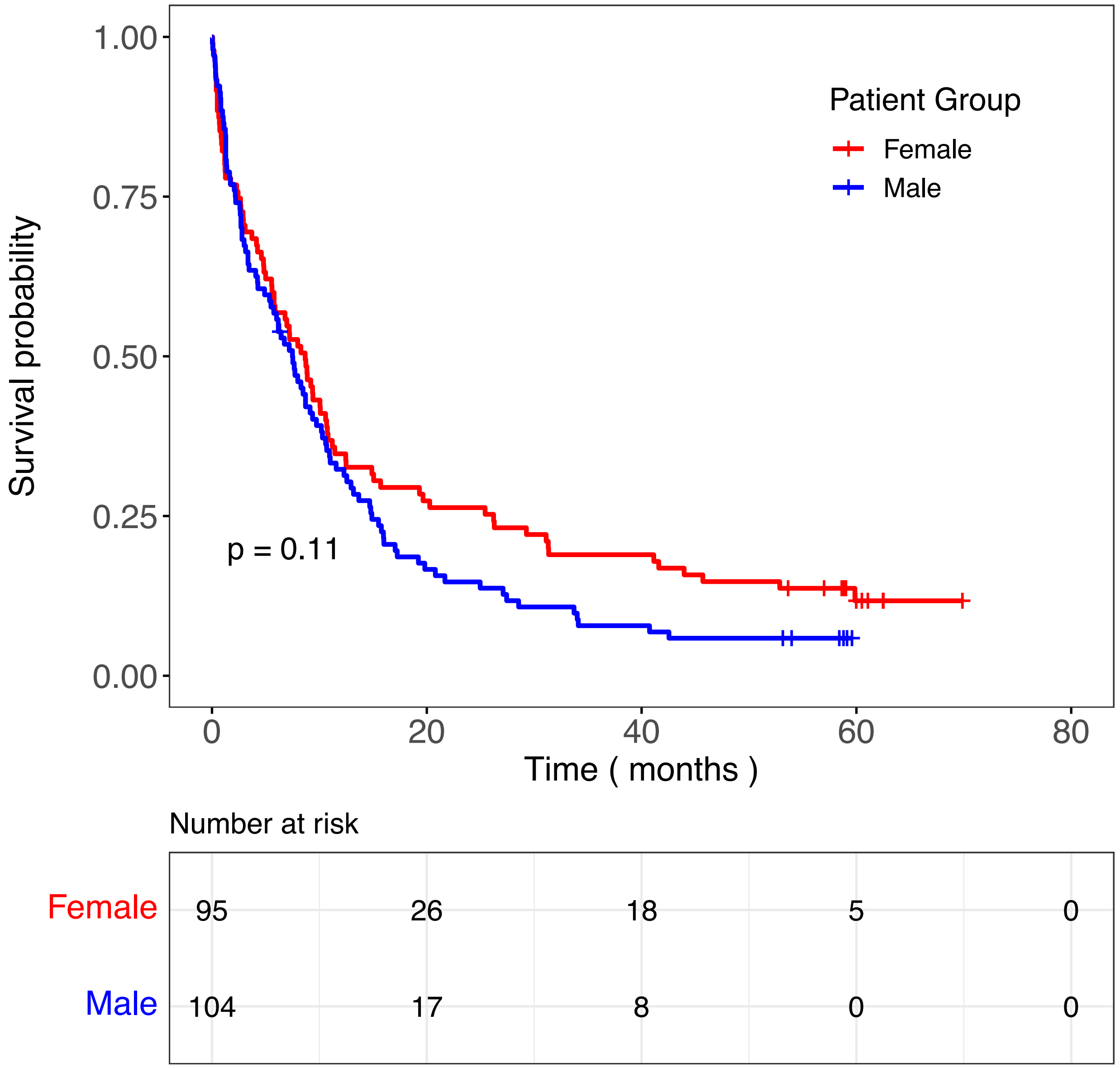

Supplementary Figure 14: Survival characteristics by sex in study cohort. Kaplan-Meier curves comparing the overall survival probabilities between all the males and females in the study cohort. P-value was calculated using the log-rank test.

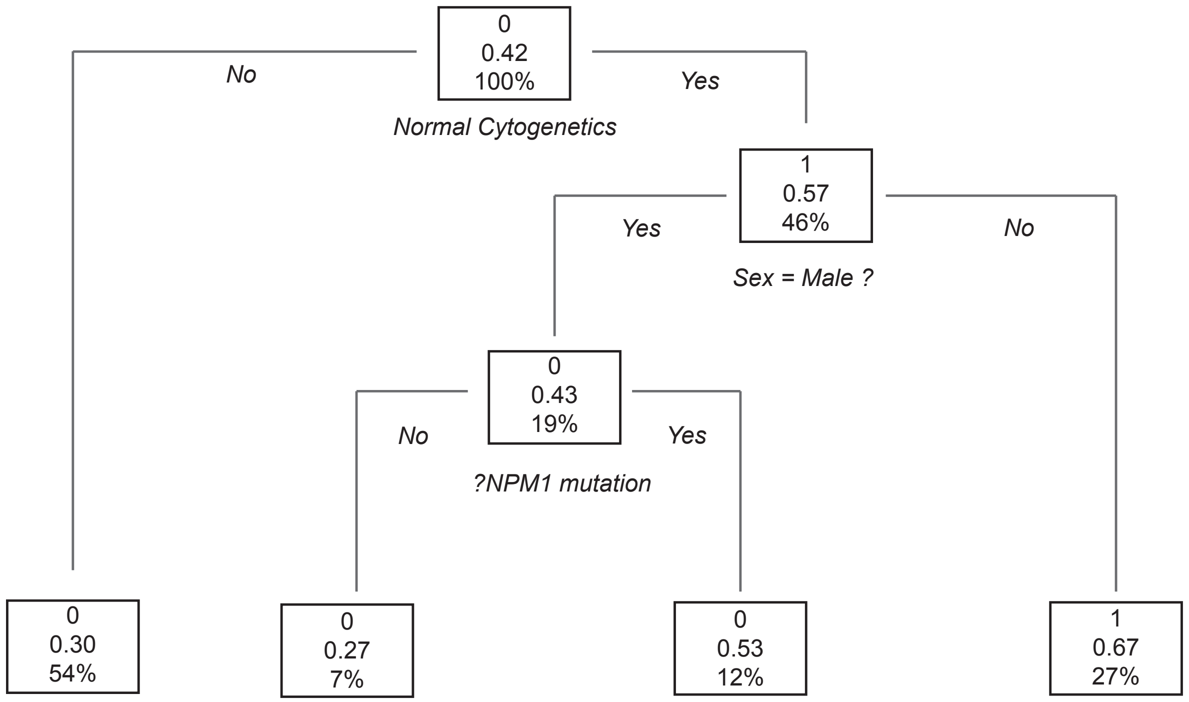

Supplementary Figure 15: Decision tree from recursive partitioning analysis for CR outcome. Decision tree from the recursive partitioning analysis identifies four distinct prognostic subgroups: Group 1) non-normal cytogenetics, Group 2) males with normal cytogenetics, Group 3) males with normal cytogenetics and NPM1 mutant status, and Group 4) females with normal cytogenetics. Groups 3 and 4 had higher probability of achieving a complete remission.

Supplementary tables and legends

Variable	Study cohort (n = 199)
**Age**	
Median (years)	69
Range (years)	60->90
**Sex: number (%)**	
Females	95 (47.7)
Males	104 (52.3)
**Disease type: number (%)**	
De novo AML	133 (66.84)
RAEB	2 (1)
RAEB-t	7 (3.52)
Secondary AML/MDS	56 (28.14)
Unknown	1 (0.5)
**Treatment: number (%)**	
Control (7+3)	92
Experimental (7+3+zosuquidar)	107
**Overall survival (months)**	
Median	7.66
Range	0.03-69.85
**Complete remission achievement: number (%)**	
Yes	85 (42.7)

No	114 (57.3)
**Diagnostic karyotype: number (%)**	
Evaluable	173 (86.9)
Not evaluable	26 (13.1)
**ELN classification : number (%)**	
Favorable	50 (23.62)
Intermediate	49 (24.63)
Unfavorable	83 (43.21)
Not evaluable	17 (8.54)

Supplementary Table 1: Study cohort characteristics. Summarized clinical features of the study cohort.

Gene	Number of patients that present a variant of unknown significance	Number of patients that present a likely oncogenic variant	Number of patients that present a known oncogenic variant	Percent of study cohort
ASXL1	1	0	25	0.13
CEBPA	2	1	16	0.1
DNMT3A	2	11	26	0.2
FLT3	1	2	14	0.085
FLT3-ITD	0	0	35	0.18
IDH1	1	0	18	0.095
IDH2	0	0	21	0.11
KRAS	0	3	11	0.07
MUC16	12	0	0	0.06
NF1	10	2	0	0.06
NPM1	0	0	59	0.3
NRAS	1	0	23	0.12
RUNX1	2	15	13	0.15
SF3B1	3	0	9	0.06
SRSF2	0	5	33	0.19
STAG2	3	12	4	0.095
TET2	3	24	23	0.25
TP53	2	6	13	0.11
TTN	17	2	0	0.095
U2AF1	1	1	18	0.1

Supplementary Table 2: Recurrent somatic mutations in the study cohort. Summarized frequencies of recurrent somatic mutations in the study cohort (n=199).

Supplementary Table 3: Somatic mutation rates comparison between study cohort and validation cohort I. For each gene mutated in at least one patient in the aAML study cohort and the independent validation cohort I, this table presents (in this order), the frequency of mutations in the aAML cohort (n = 199), the frequency of mutations in validation cohort I for patients younger than 60 years old (n = 268), the frequency of mutations in validation cohort I for patients older than 60 years old (n = 336), the significance of the frequency difference between the aAML study cohort and patients younger than 60 years old from validation cohort I (as measured by the adjusted P-value from a Fisher's exact test), and the significance of the frequency difference between the aAML study cohort and patients older than 60 years old from validation cohort I (as measured by the adjusted P-value from a Fisher's exact test).

Event	Number of patients	Percent of patients
Normal karyotype	73	0.44
Loss of 7	16	0.10
Loss of 7q	8	0.05
Loss of 5	3	0.02
Loss of 5q	16	0.10
Gain of 8	22	0.13
Loss of 17	12	0.07
Loss of 17p	3	0.02
Complex karyotype	27	0.16
Other cytogenetic abnormalities	71	0.43

Supplementary Table 4: Recurrent cytogenetic abnormalities in the study cohort.
Summarized frequencies of recurrent cytogenetics abnormalities in the study cohort (n = 166).

Cytogenetic event	Study cohort frequency	Frequency in validation cohort I (age < 60) (n=272)	Frequency in validation cohort I (age ≥ 60) (n=336)	Adjusted P-value (age < 60)	Adjusted P-value (age ≥ 60)
**Normal karyotype**	0.4397590361	0.4154411765	0.5029761905	1	1
**MLL fusion**	0.02409638554	0.1102941176	0.03273809524	0.006693360928	1
**(8/21) translocation**	0.01807228916	0.04411764706	0.005952380952	0.9088152062	1
**16 inversion**	0.01807228916	0.08823529412	0.03273809524	0.02663903411	1
**5|5q loss**	0.1144578313	0.04779411765	0.08035714286	0.09108244974	1
**7|7q loss**	0.1445783133	0.06985294118	0.119047619	0.09108244974	1
**17|17p loss**	0.09036144578	0.0625	0.05952380952	1	1
**8 gain**	0.1325301205	0.1066176471	0.1220238095	1	1
**Complex karyotype**	0.1626506024	0.1875	0.1785714286	1	1

Supplementary Table 5: Cytogenetic event rates comparison between study cohort and validation cohort I. Columns 2 to 4 are, in that order, summarized frequencies of each event in the study cohort, the younger patients (age < 60) from validation cohort I and aAML patients from validation cohort I. Columns 5 and 6 are the adjusted Wilcoxon rank-sum test P-values estimating, for each event, the statistical significance of the difference in frequencies between the

study cohort and, respectively, the younger patients from validation cohort I (column 5) and the aAML patients from validation cohort I (column 6).

Supplementary Table 6: Co-occurrence or exclusivity of the most common somatic mutations (single nucleotide variants and short insertions/deletions; n = 199). Mutations were summarized by gene, with the exception of FLT3-ITD that was independently calculated. Every gene that is mutated in >5% of the cohort (n > 10) is shown. Each cell represent the correlation between two events as measured by the R (odd columns) or the FDR (even columns).

Supplementary Table 7: Co-occurrence or exclusivity of the most common somatic events (n = 166). Mutations (single nucleotide variants and short insertions/deletions) were summarized by gene, with the exception of FLT3-ITD that was independently assessed, and each cytogenetic event was summarized at the chromosomal level, with the exception of normal and complex karyotypes (three or more unrelated chromosome abnormalities in the absence of 1 of the WHO-designated recurring translocations or inversions). Every event that is present in >5% of the cohort (n > 8) is represented. Each cell represent the correlation between two events as measured by the R (odd columns) or the FDR (even columns). Gain8 = gain of chromosome 8; del = deletion.

Supplementary Table 8: Mutation and cytogenetic events' prevalence in study cohort. Features (cytogenetic events or somatic mutations) were summarized using proportion, lower and upper 95% confidence intervals in the study cohort (n=199). del = deletion; other chr5 = chromosome 5 events other than del(5) or del(5q); other del(7) = losses of chromosome 7 other than del(7); 11q23 = chromosomal events involving 11q23; other cytogenetic events = cytogenetic event(s) other than the categories listed); IDH mutation = IDH1 or IDH2 mutations; category of somatic events were defined in "Molecular functional annotation" section in methods.

Supplementary Table 9: Univariable overall survival analysis. Results from Cox proportional hazard models were used to assess association of recurrent features with overall survival in the study cohort. Category of somatic events were defined in "Molecular functional annotation" section in methods; Sex: 1 = male and 2 = female; age was considered a continuous variable; del = deletion; trt = treatment: 1 = 'Zosuquidar' 2='Placebo'.

Supplementary Table 10: Validation cohort II features. a) Summarized features of validation cohort II. b) Molecular events frequency comparison between study cohort and validation cohort II. Factors are summarized using count and percent in validation cohort II and compared using Fisher's exact test to study cohort. Sex: 1 = male and 2 = female.

Gene	Pvalue (Proportion Test)	Confidence Interval	
		LowerLimit	UpperLimit
11q23	1	-0.100184754	0.139811793
EZH2 mutation	1	-0.100184754	0.139811793
SF3B1 mutation	1	-0.122867964	0.111212953
CBF Leukemia category	0.498264983	-0.109701789	0.27986496
IDH1 mutation	0.768153663	-0.221662911	0.107443797
KRAS mutation	1	-0.146371897	0.174343925
PHF6 mutation	1	-0.146371897	0.174343925
STAG2 mutation	0.768153663	-0.221662911	0.107443797
TP53 aneuploidy	0.763492876	-0.142112618	0.260993737
U2AF1 mutation	0.552207518	-0.254625976	0.089124811
CEBPA mutation	0.39136356	-0.286762634	0.069979418
IDH2 mutation	0.273869101	-0.318181297	0.050116029
gain chr 8	0.18930192	-0.348962932	0.029615612
NRAS mutation	0.848492278	-0.174247993	0.2814741
ASXL1 mutation	1	-0.201199886	0.205861891
DNMT3A mutation	0.745915566	-0.295686586	0.158157448
TET2 mutation	0.154161583	-0.41209925	0.029814868
RUNX1 mutation	0.955478076	-0.221885074	0.305801158
SRSF2 mutation	0.330533485	-0.425223499	0.110538184

Supplementary Table 11: Comparison of mutation and cytogenetic events between G2 and G3 patient groups. Results from proportional test estimating the difference in the frequencies of somatic mutations and cytogenetics between patients in the prognostic group G2 and G3.

Variable	Study cohort (n=497)	Low dose (n=176)	High dose (n=158)
**Age**			
Median (years)	47	46	49
Range (years)	17-59	17-59	18-59
**Treatment**			
	Fernandez, H.F., et al., Anthracycline dose intensification in acute myeloid leukemia. N Engl J Med, 2009. 361(13): p. 1249-59	Low dose standard induction (Daunorubicin+Cytarabine) with or without allotransplantation	High dose standard induction (Daunorubicin+Cytarabine) with or without allotransplantation
Sex: number (%)			
Females	241 (48.5)	85 (48.3)	75 (47.5)
Males	256 (51.5)	91 (51.7)	83 (52.5)
**Molecular criteria**			
Complex cytogenetics			
Number of cases (%)	59(11.9)	25(14.2)	27(17.01)
Unknown: number (%)	1 (0.2)	1 (0.51)	0

NPM1			
Number of mutated cases (%)	91 (18.3)	34 (19.3)	20 (12.7)
Unknown: number (%)	115(23.1)	42 (23.73)	37 (23.42)
FLT3-ITD			
Number of mutated cases (%)	108 (21.7)	46 (26.1)	30 (19)
Unknown: number (%)	42(8.43)	17 (9.6)	16(10.12)
TP53			
Number of mutated cases (%)	9 (1.8)	2 (1.13)	4 (2.5)
Unknown: number (%)	200(40.2)	78 (44.06)	64 (40.5)
**Overall survival (months)**			
Median	16.92	10.283	15.2935
Range	0.197 - 119.75	0.263 - 119.359	0.197 - 119.097
**Number of patients in G2 and G3 groups**			
G2		14	19
G3		25	26

Supplementary Table 12: NCT00049517 cohort features. Summarized features of the AML patients in the NCT00049517 cohort (younger than 60 years of age). The cohort was divided into

low dose and high dose based on the treatment regimen (see description in methods section). Factors are summarized using counts and percent in the cohort.

Supplementary Table 13: Complete remission (CR) analysis. A) Percent of population who achieved CR. B) Logistic regression was used to assess association of recurrent features with CR. Age was considered a continuous variable; trt = treatment: 1 = 'Zosuquidar' 2='Placebo'; del = deletion; category of somatic events were defined in “Molecular functional annotation” section in methods.

Sequencing variable	Median (tumor samples)	MAD (tumor samples)	Median (germline samples)	MAD (germline samples)
**Mean target coverage**	94.6	7.83	91.6	8.12
**Percentage of target bases covered at 1x**	99.8	7.80×10^{-4}	99.8	7.90×10^{-4}
**Percentage of target bases covered at 10x**	98.7	4.91×10^{-3}	98.6	5.59×10^{-3}
**Percentage of target bases covered at 50x**	80.8	6.31×10^{-2}	80.5	4.51×10^{-2}

Supplementary Table 14: Summary statistics for the whole exome sequencing. Median and median absolute deviation (MAD) for mean target coverage and the percentages of target bases covered at 1x, 10x and 50x for both tumor and germline samples.

References

1 Cripe LD, Uno H, Paietta EM, Litzow MR, Ketterling RP, Bennett JM *et al.* Zosuquidar, a novel modulator of P-glycoprotein, does not improve the outcome of older patients with newly diagnosed acute myeloid leukemia: a randomized, placebo-controlled trial of the Eastern Cooperative Oncology Group 3999. *Blood* 2010; **116**: 4077–4085.

2 Li H. Aligning sequence reads, clone sequences and assembly contigs with BWA-MEM. 2013.http://arxiv.org/abs/1303.3997 (accessed 28 Aug2017).

3 Cingolani P, Platts A, Wang LL, Coon M, Nguyen T, Wang L *et al.* A program for annotating and predicting the effects of single nucleotide polymorphisms, SnpEff: SNPs in the genome of Drosophila melanogaster strain w1118; iso-2; iso-3. *Fly (Austin)* 2012; **6**: 80–92.

4 Lek M, Karczewski KJ, Minikel E V., Samocha KE, Banks E, Fennell T *et al.* Analysis of protein-coding genetic variation in 60,706 humans. *Nature* 2016; **536**: 285–291.

5 Papaemmanuil E, Gerstung M, Bullinger L, Gaidzik VI, Paschka P, Roberts ND *et al.* Genomic Classification and Prognosis in Acute Myeloid Leukemia. *N Engl J Med* 2016; **374**: 2209–2221.

6 Szankasi P, Jama M, Bahler DW. A new DNA-based test for detection of nucleophosmin exon 12 mutations by capillary electrophoresis. *J Mol Diagn* 2008; **10**: 236–241.

7 Andersson AK, Miller DW, Lynch JA, Lemoff AS, Cai Z, Pounds SB *et al.* IDH1 and IDH2 mutations in pediatric acute leukemia. *Leukemia* 2011; **25**: 1570–1577.

8 Kiyoi H, Naoe T, Yokota S, Nakao M, Minami S, Kuriyama K *et al.* Internal tandem duplication of FLT3 associated with leukocytosis in acute promyelocytic leukemia. Leukemia Study Group of the Ministry of Health and Welfare (Kohseisho). *Leukemia* 1997; **11**: 1447–1452.

9 Noguera NI, Breccia M, Divona M, Diverio D, Costa V, De Santis S *et al.* Alterations of the FLT3 gene in acute promyelocytic leukemia: association with diagnostic characteristics and analysis of clinical outcome in patients treated with the Italian AIDA protocol. *Leukemia* 2002; **16**: 2185–2189.

10 Ley TJ, Miller C, Ding L, Raphael BJ, Mungall AJ, Robertson AG *et al.* Genomic and epigenomic landscapes of adult de novo acute myeloid leukemia. *N Engl J Med* 2013; **368**: 2059–2074.

11 Lindsley RC, Mar BG, Mazzola E, Grauman P V, Shareef S, Allen SL *et al.* Acute myeloid leukemia ontogeny is defined by distinct somatic mutations. *Blood* 2015; **125**: 1367–1376.

12 Grimwade D, Ivey A, Huntly BJP. Molecular landscape of acute myeloid leukemia in younger adults and its clinical relevance. *Blood* 2016; **127**: 29–41.

13 Eisfeld A-K, Mrózek K, Kohlschmidt J, Nicolet D, Orwick S, Walker CJ *et al.* The mutational oncoprint of recurrent cytogenetic abnormalities in adult patients with de novo acute myeloid leukemia. *Leukemia* 2017; **31**: 2211–2218.

14 Rahmani NE, Ramachandra N, Bhagat TD, Gordon S, Pradhan K, Rivera Pena B *et al.* ASXL1 Mutations Are Associated with Widespread and Distinct DNA Methylation Alterations. *Blood* 2019; **134**: 2989.

15 Desai P, Mencia-Trinchant N, Savenkov O, Simon MS, Cheang G, Lee S *et al.* Somatic mutations precede acute myeloid leukemia years before diagnosis. *Nat Med* 2018; **24**: 1015–1023.

16 Bolger AM, Lohse M, Usadel B. Trimmomatic: a flexible trimmer for Illumina sequence data. *Bioinformatics* 2014; **30**: 2114–2120.

17 Faust GG, Hall IM. SAMBLASTER: fast duplicate marking and structural variant read extraction. *Bioinformatics* 2014; **30**: 2503–2505.

18 Lai Z, Markovets A, Ahdesmaki M, Chapman B, Hofmann O, McEwen R *et al.* VarDict: a novel and versatile variant caller for next-generation sequencing in cancer research. *Nucleic Acids Res* 2016; **44**: e108.

19 McLaren W, Gil L, Hunt SE, Riat HS, Ritchie GRS, Thormann A *et al.* The Ensembl Variant Effect Predictor. *Genome Biol* 2016; **17**: 122.

20 Sherry ST, Ward MH, Kholodov M, Baker J, Phan L, Smigielski EM *et al.* dbSNP: the NCBI database of genetic variation. *Nucleic Acids Res* 2001; **29**: 308–311.

21 Canisius S, Martens JWM, Wessels LFA. A novel independence test for somatic alterations in cancer shows that biology drives mutual exclusivity but chance explains most co-occurrence. *Genome Biol* 2016; **17**: 261.

22 Tyner JW, Tognon CE, Bottomly D, Wilmot B, Kurtz SE, Savage SL *et al.* Functional genomic landscape of acute myeloid leukaemia. *Nature* 2018; **562**: 526–531.

23 Breiman L, Friedman J, Stone CJ, Olshen R. *Classification and Regression Trees*. Chapman and Hall/CRC, 1984.

24 Döhner H, Estey E, Grimwade D, Amadori S, Appelbaum FR, Büchner T *et al.* Diagnosis and management of AML in adults: 2017 ELN recommendations from an international expert panel. *Blood* 2017; **129**: 424–447.

25 Gönen M, Heller G. Concordance Probability and Discriminatory Power in Proportional Hazards Regression. *Biometrika* 2005; **92**: 965–970.

26 Rapaport F, Neelamraju Y, Baslan T, Hassane D, Gruszczynska A, Robert de Massy M *et al.* Genomic and evolutionary portraits of disease relapse in acute myeloid leukemia. *Leukemia* 2021; **35**: 2688–2692.

27 Foran JM, Sun Z, Claxton DF, Lazarus HM, Thomas ML, Melnick A *et al.* North American Leukemia, Intergroup Phase III Randomized Trial of Single Agent Clofarabine As Induction and Post-Remission Therapy, and Decitabine As Maintenance Therapy in Newly-Diagnosed Acute Myeloid Leukemia in Older Adults (Age ≥60 Years): A Trial of. *Blood* 2015; **126**: 217.

28 Patel JP, Gönen M, Figueroa ME, Fernandez H, Sun Z, Racevskis J *et al.* Prognostic relevance of integrated genetic profiling in acute myeloid leukemia. *N Engl J Med* 2012; **366**: 1079–1089.

29 Fernandez HF, Sun Z, Yao X, Litzow MR, Luger SM, Paietta EM *et al.* Anthracycline dose intensification in acute myeloid leukemia. *N Engl J Med* 2009; **361**: 1249–1259.
